# A Global Perspective on Pyrazinamide Resistance: Systematic Review and Meta-Analysis

**DOI:** 10.1371/journal.pone.0133869

**Published:** 2015-07-28

**Authors:** Michael G. Whitfield, Heidi M. Soeters, Robin M. Warren, Talita York, Samantha L. Sampson, Elizabeth M. Streicher, Paul D. van Helden, Annelies van Rie

**Affiliations:** 1 SA MRC Centre for TB Research, Stellenbosch University, South Africa; 2 DST/NRF Centre of Excellence for Biomedical TB Research, Stellenbosch University, South Africa; 3 Division of Molecular Biology and Human Genetics, Stellenbosch University, South Africa; 4 Faculty of Medicine and Health Sciences, Stellenbosch University, South Africa; 5 Department of Epidemiology, University of North Carolina at Chapel Hill, Chapel Hill, North Carolina, United States of America; 6 International Health Unit, Epidemiology and Social Medicine, Faculty of Medicine, University of Antwerp, Antwerp, Belgium; St. Petersburg Pasteur Institute, RUSSIAN FEDERATION

## Abstract

**Background:**

Pyrazinamide (PZA) is crucial for tuberculosis (TB) treatment, given its unique ability to eradicate persister bacilli. The worldwide burden of PZA resistance remains poorly described.

**Methods:**

Systematic PubMed, Science Direct and Scopus searches for articles reporting phenotypic (liquid culture drug susceptibility testing or pyrazinamidase activity assays) and/or genotypic (polymerase chain reaction or DNA sequencing) PZA resistance. Global and regional summary estimates were obtained from random-effects meta-analysis, stratified by presence or risk of multidrug resistant TB (MDR-TB). Regional summary estimates were combined with regional WHO TB incidence estimates to determine the annual burden of PZA resistance. Information on single nucleotide polymorphisms (SNPs) in the *pncA* gene was aggregated to obtain a global summary.

**Results:**

Pooled PZA resistance prevalence estimate was 16.2% (95% CI 11.2-21.2) among all TB cases, 41.3% (29.0-53.7) among patients at high MDR-TB risk, and 60.5% (52.3-68.6) among MDR-TB cases. The estimated global burden is 1.4 million new PZA resistant TB cases annually, about 270,000 in MDR-TB patients. Among 1,815 phenotypically resistant isolates, 608 unique SNPs occurred at 397 distinct positions throughout the *pncA* gene.

**Interpretation:**

PZA resistance is ubiquitous, with an estimated one in six incident TB cases and more than half of all MDR-TB cases resistant to PZA globally. The diversity of SNPs across the *pncA* gene complicates the development of rapid molecular diagnostics. These findings caution against relying on PZA in current and future TB drug regimens, especially in MDR-TB patients.

## Introduction

The global burden of tuberculosis (TB) remains a major concern for health authorities worldwide. In 2013, there were an estimated 9.0 million new cases and 1.5 million deaths from TB [1]. Treatment regimens for drug-susceptible TB consist of rifampicin (RIF), isoniazid (INH), pyrazinamide (PZA) and ethambutol (EMB). PZA forms a critical cornerstone of this regimen given its unique ability to eradicate persister bacilli, which allowed treatment shortening from 9–12 months to 6 months [2,3]. PZA will likely remain an important component of treatment regimens for drug-susceptible and multidrug-resistant TB (MDR-TB) because of its distinctive mode of action (interference with ATP production) [4,5] and its synergistic pharmacokinetic properties with two of the new anti-TB drugs: the diarylquinoline Bedaquiline (affects F1F0 proton ATP synthase) and the nitroimidazole PA-824 (enhances PZA activity by altering the cell wall integrity) [6–12].

PZase is only active at low pH (pH 5.00–6.00) as experienced in the phagosomal compartment. Down-regulation of efflux pumps in the persister *Mycobacterium tuberculosis* results in intracellular accumulation of POA, which leads to the depletion of membrane potential [13–17], and inhibits trans-translation [5]. The decreasing membrane potential is detrimental to non-replicating persisters whose energy requirements are finely balanced [9]. Trans-translation plays a role for stress survival and pathogenesis as it aids the management of stalled ribosomes, damaged mRNA and proteins during stressful conditions [14, 18–20].

Even though PZA is a crucial component of TB treatment, little is known about the prevalence of PZA resistance, particularly on a global scale. PZA drug susceptibility testing (DST) is technically challenging and rarely performed as part of routine care or routine drug surveillance in resource-limited settings. Two phenotypic PZA DSTs, BACTEC 460TB and BACTEC MGIT 960 (Becton Dickinson, Sparks, MD) exist; only BACTEC MGIT 960 is currently commercially available. Neither of these assays has been approved by the World Health Organization (WHO), likely due to their complexity and inconsistency, with frequent false positive results [21,22]. Classic and modified Wayne’s PZase methods, which assess the function of the PZase enzyme based on a colorimetric change at critical concentrations of 100μg/ml to 400μg/ml, respectively [23], are also not endorsed by the WHO. More recently, genotypic PZA assays have been developed based on observations that mutations in the *pncA* gene are the primary mechanism of PZA resistance [15,24–27]. The *pncA* gene encodes the pyrazinamidase (PZase) enzyme, which converts PZA, a pro-drug, into the active pyrazinoic acid (POA) [14,24]. These molecular techniques, albeit not approved by WHO, are the most commonly used techniques in PZA drug resistance studies.

Understanding regional differences in PZA resistance and its causal mutations is important for policy decisions regarding treatment regimens for drug-resistant TB and development of sequence-based diagnostics [26]. The aims of this review were to summarize the prevalence of PZA resistance globally and by WHO geographic regions, and to estimate the annual burden of PZA resistance, both stratified by MDR-TB status. We also summarize the global frequency and distribution of single nucleotide polymorphisms (SNPs) in the *pncA* gene in PZA resistant isolates.

## Materials and Methods

### Search Strategy and Selection Criteria

We followed the Preferred Reporting Items for Systematic Reviews and Meta-Analyses (PRISMA) guidelines.[28] We searched PubMed, ScienceDirect and Scopus on July 14, 2014 for relevant articles published in the English language between 1998 and 2014 using an *a priori* protocol. The search terms “tuberculosis AND (pyrazinamide OR PZA) AND (phenotype OR genotype OR PZase OR pyrazinamidase OR *pncA* OR BACTEC OR mutations OR resistance OR resistant OR susceptibility OR sequence analysis OR microbial sensitivity tests OR molecular typing)” were used to identify articles reporting on PZA resistance using any of the methods of interest: phenotypic PZA DST, PZase activity assays, and/or genotypic PZA assays. Additional articles were identified from reference lists and review articles.

Studies were eligible for inclusion in the meta-analysis of prevalence of PZA resistance if (1) PZA DST was assessed using at least one the following phenotypic tests: BACTEC liquid-based DST 460 or 960, considered the reference standard for PZA DST, or PZase activity assays using classic or modified Wayne’s methods [24]. If both BACTEC 960 and BACTEC 460 results were reported, only the BACTEC 960 results were included. BACTEC 960 results were preferred to the BACTEC 460 due to the BACTEC 460 no longer being commercially available. If results from BACTEC 460 using both a PZA concentration of 100μg/mL and 50μg/mL were reported, the results using 100μg/mL were included. If results from both classical and modified Wayne’s PZase assays were reported, the classical Wayne’s results were included. Authors were contacted if no clear method of PZA DST is described in the article. To be eligible for inclusion in the analysis, studies had to provide information on the MDR-TB risk status (patients diagnosed with MDR-TB, patients at high-risk of MDR-TB, or inclusion of any TB case), reporting on a single subgroup or stratifying results by subgroup. High-risk of MDR-TB was defined as an isolate being resistant to at least one anti-TB drug. Any TB was defined as the inclusion of patients independent of drug resistance profile.

Studies were eligible for inclusion in the descriptive SNP analysis if they performed genotypic testing using polymerase chain reaction (PCR) and DNA sequencing and characterized the found SNPs.

For both the PZA prevalence and *pncA* SNP analysis, studies including samples from multiple countries were only included if the results were stratified by country. In studies collecting multiple samples from a single patient, only the first sample result was used. Where a study performed repeat testing on a sample, only the first result was retained in the review. No additional exclusion criteria were imposed.

### Data Extraction

MGW and RMW independently reviewed titles and abstracts of original studies retrieved by the search. MGW and TY reviewed full-text and references of selected articles. MGW and HMS abstracted study data from full reports.

The following information, if available, was abstracted from each article: first author surname; publication year; WHO region (Africa, Americas, Eastern Mediterranean, Europe, South East Asia, or Western Pacific); study dates; study design; study setting; sample size; MDR risk subgroup; age; gender; HIV status; exclusion criteria; specimen type; phenotypic DST method; PZase activity assay; genotypic method; and whether the up and down stream regions of *pncA* were sequenced. The number of patients with PZA resistance according to liquid DST, the lack of PZase activity, or genotypic mutations in the *pncA* gene was also recorded. As Taiwan was not defined by the WHO, it was grouped in the Western Pacific region.

### Statistical Analysis

Summary estimates for the prevalence of PZA resistance, calculated using random-effects meta-analytic methods in STATA 13 (StataCorp LP, College Station, TX), were determined globally and for each WHO region, stratified according to whether the subgroup of patients had MDR-TB, were at high-risk of MDR-TB, or had any TB.

Estimation of the burden of PZA resistant TB, stratified by region and by presence of MDR-TB were obtained by multiplying the regional point estimates obtained by the random-effects meta-analysis by the most recent (2011) regional WHO estimates for incident TB and MDR-TB cases [1].

The analysis of SNPs in the *pncA* gene was descriptive. We present results according to nucleotide position in order to identify regions of SNP clustering within the *pncA* gene. In addition, we present a detailed description of all SNPs reported, including the location and type of polymorphism and countries where this SNP has been isolated, as well as whether this SNPs has been linked to a resistant phenotype only or has been observed in both resistant and susceptible isolates, with phenotypic resistance defined by BACTEC DST results or the PZase enzyme assay results if BACTEC DST result was not available.

Role of the funding source: The funders of the study (NIH and NRF) had no role in study design, data collection, data analysis, data interpretation, or writing of the report. The corresponding author had full access to all data in the study and had final responsibility for the decision to submit for publication.

## Results

### Selected Studies

**Fig 1:**
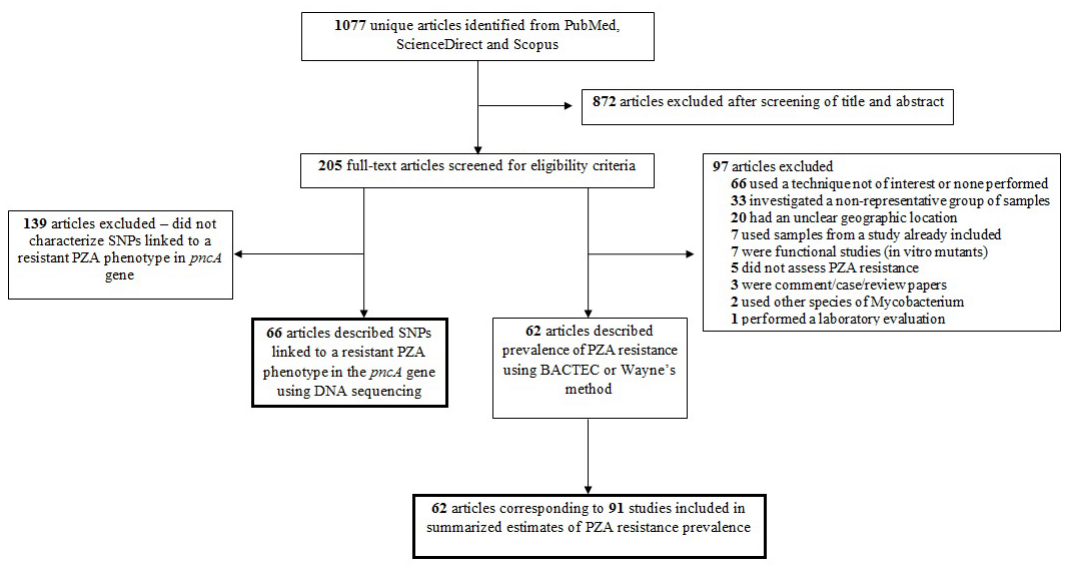
Flow diagram describing article selection.

The literature search resulted in 1077 abstracts identified. Of these, 205 full-text articles were selected for review. In total, 62 [29–90] articles met the eligibility criteria for reporting PZA resistance, resulting in 91 datasets due to articles having isolates from multiple WHO regions as well as isolates which met different cohort type criteria (Fig 1). Of the 205 full-text articles reviewed, 66 [27,59–123] articles were eligible for inclusion in the analysis of SNP frequency and distribution.

### Phenotypic PZA Resistance: Study and Population Characteristics

**Fig 2:**
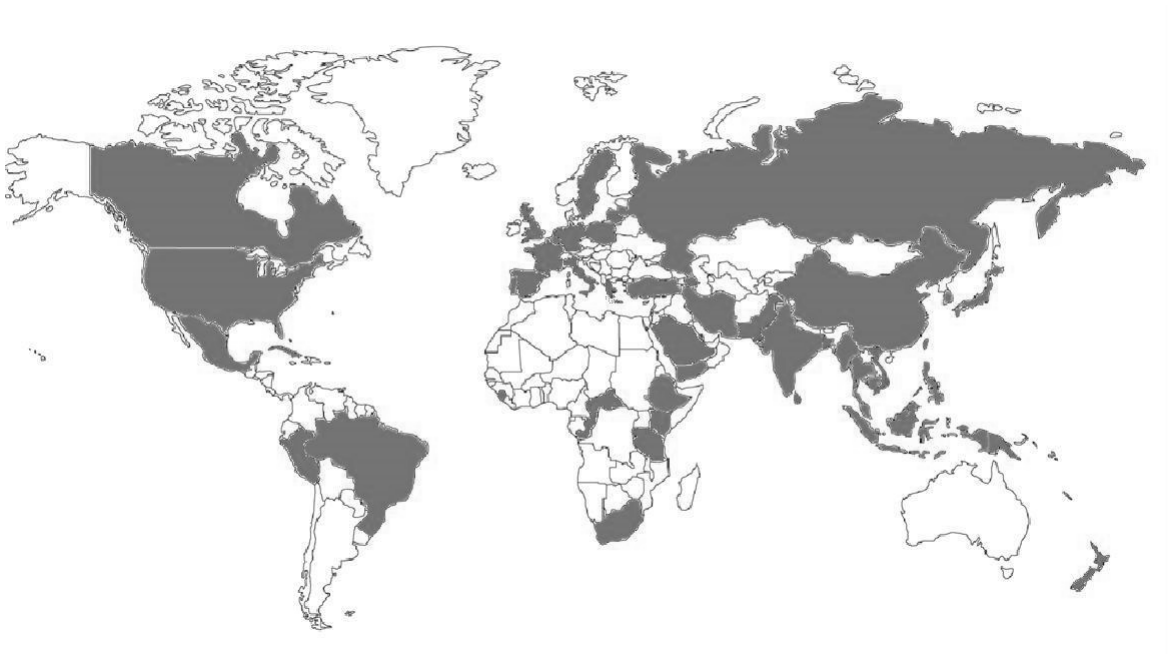
Global distribution of included studies. Countries are shaded if a study was included in this review.

The 62 [29–90] final studies provided phenotypic PZA resistance data on 35,950 *M*. *tuberculosis* clinical isolates. According to WHO regions, 8 [39,59,60,68,69,78] studies were from African region, 20 [29,30,38,40,41,43–47,59,70–72,79–83] from the Americas, 3 [31,48,80] from the Eastern Mediterranean, 20 [32,33,49–54,59,61,62,84–86] from European, 17 [34–36,47,55,56,59,63,73,80] from South East Asia, and 23 [37,42,57,58,64–67,74–77,80,86–90] from the Western Pacific region (Fig 2). Most (53/91) [30,35,36,39–61,63,64,78–90] estimates of PZA prevalence were provided for studies including any TB patient, independent of drug resistance profile; 25 [29–37,42,59–67] studies reported PZA resistance among individuals with confirmed MDR-TB, and 13 [33,34,38,68–77] estimates were available for individuals at high-risk of MDR-TB. Study and population characteristics are displayed in S1 Table.

### Phenotypic PZA Resistance: Regional and Pooled Prevalences and Annual Burden

**Fig 3:**
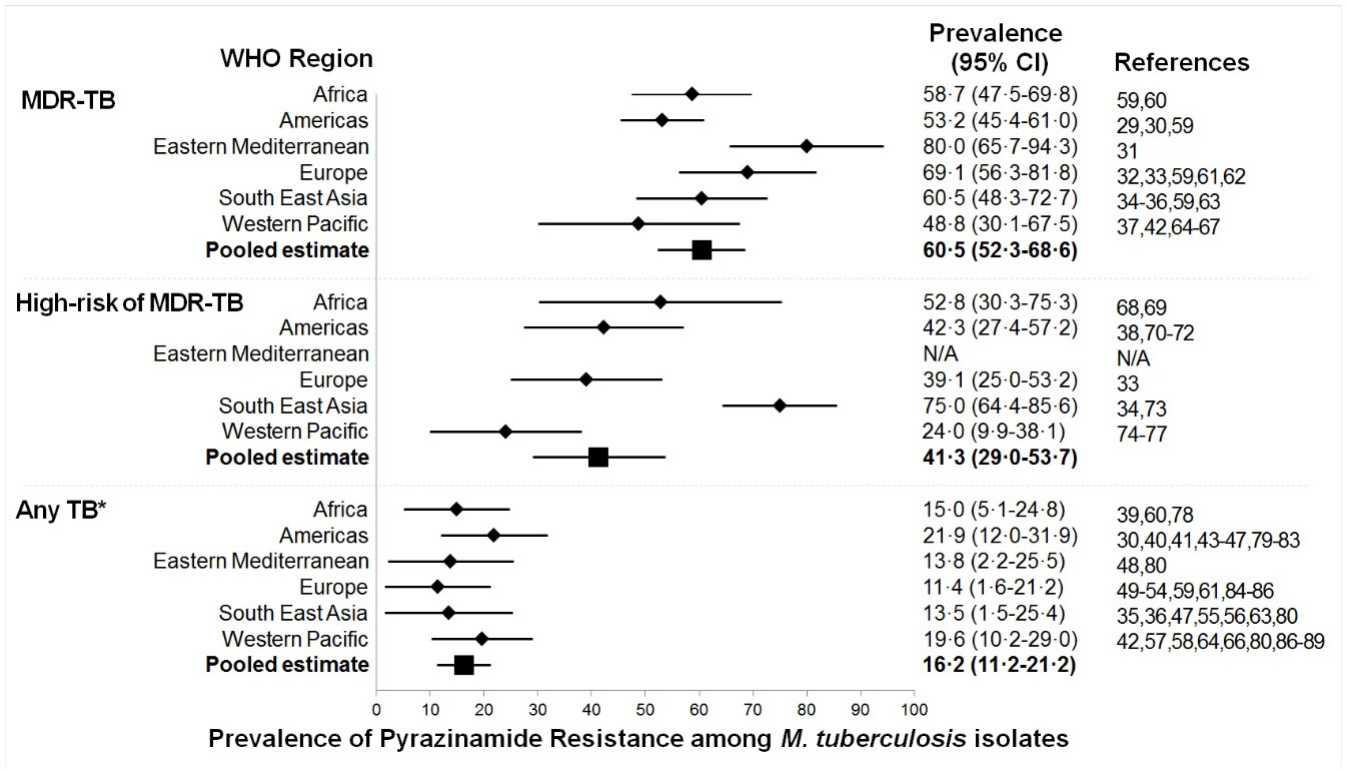
Forest plot for the summary estimates of pyrazinamide prevalence by WHO region and presence or risk of MDR-TB. Abbreviations: CI, confidence interval; DST, drug susceptibility test; MDR-TB, multi-drug resistant tuberculosis; N/A, not applicable; WHO, world health organization. MDR-TB was defined as an isolate being resistant to RIF and INH. High risk of MDR-TB was defined as an isolate being resistant to at least one anti-TB drug. *Any TB was defined as the inclusion of patients independent of drug resistance profile.

PZA resistance is prevalent across the entire globe and has been reported in all six WHO-defined regions (Fig 3). The pooled summarized prevalence estimate of PZA resistance was 60.5% (95% CI 52.3–68.6%) in MDR-TB patients, 41.3% (95% CI 29.0–53.7%) in TB patients at high-risk of MDR-TB, and 16.2% (95% CI 11.2–21.2%) in studies including any TB patient irrespective of resistance profile. In all six WHO regions, the prevalence of PZA resistance was two to six times higher in MDR-TB patients compared to the population of all TB patients.

PZA resistance prevalence among cases of MDR-TB ranged from 48.8% (95% CI 30.1–67.5%) in the Western Pacific to 80.0% (95% CI 65.7–94.3%) in the Eastern Mediterranean, but the latter estimate was based on a single study [31]. The estimated PZA prevalence among those at high risk of MDR-TB varied greatly, from 24.0% (95% CI 9.9–38.1%) in the Western Pacific region to 75.0% (95% CI 64.4–85.6%) in South East Asian region. In the general TB population, PZA prevalence estimated ranged from 11.4% (95% CI 1.6–21.2) in the European region to 21.9% (95% CI 12.0–31.9%) in the Americas.

Multiplying the regional WHO estimates for the annual number of new TB cases and incident MDR-TB cases by the pooled summarized prevalence estimates of PZA resistance, we estimated that about 1.4 million PZA resistant TB cases occur annually, corresponding to 16.2% of the 9.0 million incident TB cases in 2013 (Table 1). Of these, an estimated 270,000 occur in people also resistant to at least isoniazid and rifampicin, representing 60% of all incident cases of MDR-TB estimated in 2013.

**Table 1:** Estimated annual burden of new PZA resistant tuberculosis cases, overall and among MDR-TB patients, globally and by WHO region.

WHO region	Incident TB cases*	Incident PZA resistant cases	Incident MDR-TB cases*	Incident PZA resistant MDR-TB cases
African	2,600 000	416,000	78,000	45,800
Americas	280,000	44,800	8,400	4,468
Eastern Mediterranean	750,000	120,000	27,000	21,600
European	360,000	57,600	91,000	62,881
South East Asian	3,400 000	544,000	135,000	81,675
Western Pacific	1,600 000	256,000	125,000	61,000
**GLOBAL**	9,000 000	1,438 000	464,400	277,424

Abbreviations: MDR-TB, multi-drug resistant tuberculosis; PZA, pyrazinamide; TB, tuberculosis; WHO, World Health Organization.

* Incidence of TB cases from the World Health Organization Global Tuberculosis Report 2014.

### Single Nucleotide Polymorphism Distribution in *pncA* Gene

**Fig 4:**
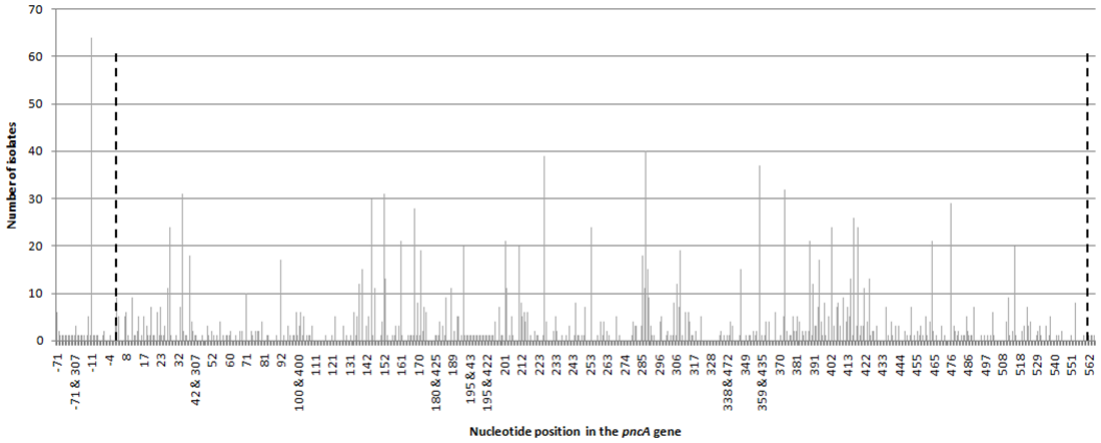
Distribution of reported single nucleotide polymorphisms (SNPs) throughout the *pncA* gene. Dashed lines indicate the open reading frame for the *pncA* gene.

The 66 [27,59–123] articles provided SNP data from 8,651 *M*. *tuberculosis* clinical isolates. According to WHO region, five [60,68,69,78,102] articles were from Africa, 14 [27,70–72,79,81–83,97,100,105,107,114,120] from the Americas, two [80,92] from the Eastern Mediterranean, 17 [61,62,84–86,93,96,103,104,106,108–110,115–117,119] from Europe, six [59,63,73,118,121,123] from South East Asia, and 22 [64–67,74–77,87–91,94,95,98,99,101,111–113,122] from the Western Pacific. A SNP in the *pncA* region was detected in the 1,815 of the 8,651 isolates, with 608 unique polymorphisms in 397 positions in the gene (S2 Table). SNPs were found throughout the entire *pncA* gene and flanking region with no particular clustering or hot spots (Fig 4). There are however, a few SNPs which were found to be more frequently than others such as -11 and 195, but even the 20 most frequent SNPs only represented one third of all isolates with phenotypic PZA resistance.

## Discussion

*M*. *tuberculosis* resistance to rifampicin and isoniazid is well described and monitored, either through continuous surveillance or periodic surveys of a representative sample of patients [124]. In contrast, resistance to ethambutol and PZA, the other two front line drugs, is not routinely monitored and thus poorly described. In this systematic review and meta-analysis, we found that PZA resistance is ubiquitous and increases in prevalence as risk of resistance to other drugs increases, with pooled summary estimates for the prevalence of PZA resistance of 16.2% in the total population of TB patients, 41.3% among TB patients at high risk of MDR-TB, and 60.5% in patients with confirmed MDR-TB. The high prevalence of PZA resistance results in an annual estimated burden of 1.4 million new cases of PZA resistant TB, of which about 270,000 occur in patients with MDR-TB [1]. This high prevalence of PZA resistance observed in all regions of the world and across different TB patient groups is an important finding as PZA is not only a key component of all current regimens for both drug susceptible and drug resistant TB but is also included in *all* novel drug regimens currently undergoing evaluation in clinical phase II or III trials for treatment of drug-susceptible or drug-resistant TB [125].

While our review aimed to comprehensively summarize information on PZA as a global public health problem, a different systematic review by Chang *et al* aimed to summarize the performance of molecular and PZase assays compared to culture-based phenotypic DST [126]. In that review, the median (range) of PZA resistance was 5% (0% to 9%) in non-MDR isolates and 51% (31% to 89%) in MDR *M*. *tuberculosis* isolates [126]. Our pooled prevalence estimates were higher than the median prevalence reported by Chang *et al*., especially in the overall TB patient population (16.2%). The summary estimate in our review may have overestimated the overall prevalence of PZA resistance among TB patients if the proportion of patients with drug resistance included in the studies was higher than that observed in the general population of the country where the studies took place.

The high prevalence of PZA resistance and its inclusion in both standard and novel drug regimens highlights the need for routine PZA resistance testing. Others have suggested that molecular assays may be the way forward for detecting PZA resistance, based on findings that molecular assays targeting *pncA* can detect PZA resistance in MDR-TB isolates with high positive predictive values and rule out PZA resistance in non-MDR isolates with high negative predictive values [126,127]. However, DNA sequencing studies have revealed that mutations and/or polymorphisms occur across the entire length of the *pncA* gene, suggesting that sequencing the entire *pncA* gene would be essential to capture all possible mutations [109,117,127–129]. In this review, we confirmed that on a global scale, SNPs are distributed throughout the entire *pncA* gene. Whereas the tbdream database [14,26] previously reported on 278 unique polymorphisms prior to 2009, our systematic review provides an updated overview, with information on more than 600 unique polymorphisms in approximately 400 positions in *pncA* (including the upstream flanking region). A few SNPs occurred more frequently than others, possibly because these SNPs are rooted in ancestral strains. Consequently, developing a molecular assay to detect PZA resistance will be much more challenging compared to other genes (*rpoB*, *gyrA*, *embB*) which have been found to have clear resistance-causing hot spots [130]. The identification of causal PZA mutations is further complicated by the fact that not all non-synonymous mutations cause phenotypic resistance [80] and that mutations in the *pncA* gene can be absent in a small percentage of phenotypically PZA resistant isolates, [66,102] suggesting that PZA resistance could be conferred via an alternative mechanisms such as mutations in the *rpsA* gene [81]. Whereas development of simplified micro-array systems for simultaneous detection of rifampicin, isoniazid and ethambutol resistance may be possible, [131] inclusion of assessment of PZA resistance may thus require a different approach such as targeted DNA sequencing or next generation sequencing [127,132].

A major strength of our study was the comprehensive inclusion of studies from across the globe and stratification of estimates by region and TB patient category. Insight into PZA prevalence by region and TB patient category provides essential information for the development and clinical use of future PZA resistance tests. Our study adds to the review by Chang *et al*, which was aimed at summarizing PZA resistance assay performance, not PZA resistance prevalence. Our review also complements the recent study by Miotto *et al*, which presented *pncA* sequence results of 1950 clinical isolates obtained from multiple laboratories, but did not stratify results by regions or MDR-TB status [127]. Our review was however limited by the data quality of the original studies [126]. Misclassification of PZA resistance may have occurred in the studies included, and due to false positive results, may have resulted in an overestimate of the true prevalence of PZA resistance. Phenotypic PZA drug susceptibility testing has not been endorsed by the WHO perhaps due to concerns surrounding false positivity related to the acidity of the media, inoculum size and critical concentration used [17,133,134]. Alternative techniques, the PZase activity assays, [23,135] have been used in the hope of identifying PZA resistance more accurately, but the interpretation of colourmetric change for these assays is highly subjective [56]. It is uncertain whether all mutations observed in the *pncA* region are associated with resistance. Similar to the tbdream database approach, [26] we chose not to make *a priori* decisions as to whether mutations described actually confer resistance and report any mutation found in a PZA-resistant isolate. Another limitation was the restricted number of studies for certain regions (especially Eastern Mediterranean, where all strains included came from a single study in Pakistan) and the lack of adequate representation of countries within certain regions (especially for Africa, where almost all isolates included came from South Africa, and the Americas). This not only resulted in uncertainty of the accuracy of the point estimates and wide confidence intervals but also highlights the lack of information on PZA resistance in several regions of the world. Finally, many studies did not provide clinical information and we were therefore unable to stratify our analysis by new versus re-treatment status.

The ubiquitous presence of PZA resistance is of global interest and should signal a call to action. Development of rapid diagnostics to detect PZA resistance will be essential to maximize the efficacy of novel treatment regimens and minimize the risk of development of resistance to novel drugs. In addition, the high prevalence of PZA resistance, especially among MDR-TB patients, highlights the need for development of treatment regimens that can be effectively used in patients with PZA-resistant MDR-TB.

## Supporting Information

S1 PRISMA Checklist PRISMA check list for meta analysis (http://www.prisma-statement.org/2.1.2%20-%20PRISMA%202009%20Checklist.pdf). (PDF)

S1 Table Additional study and population characteristics. Abbreviations: MDR-TB, multi-drug resistant tuberculosis; PZA, pyrazinamide; TB, tuberculosis; WHO, World Health Organization; lab, laboratory; N/A, not applicable; N/S, not stated; HR-MDR, high-risk multi-drug resistant tuberculosis; MTB, *Mycobacterium tuberculosis;* PCR, polymerase chain reaction, East Med, Eastern Mediterranean.
(PDF)

S2 Table Single-nucleotide polymorphisms (SNPs) detected in the *pncA* gene, by country and resistance phenotype. Abbreviations: A, adenine; bp, base pair; C, cytosine; del, deletion; G, guanine; R, resistant; SNP, single-nucleotide polymorphism; T, thymine. * Article found one isolate sensitive and one isolate resistant.
(PDF)
