# Supplementary material for: A Global Perspective on Pyrazinamide Resistance: Systematic Review and Meta-Analysis
Source: PLoS One. 2015 Jul 28;10(7):e0133869. doi: 10.1371/journal.pone.0133869 (PMC4517823; doi:10.1371/journal.pone.0133869)
Supplement: S1 Table — Abbreviations: MDR-TB, multi-drug resistant tuberculosis; PZA, pyrazinamide; TB, tuberculosis; WHO, World Health Organization; lab, laboratory; N/A, not applicable; N/S, not stated; HR-MDR, high-risk multi-drug resistant tuberculosis; MTB, Mycobacterium tuberculosis; PCR, polymerase chain reaction, East Med, Eastern Mediterranean. (PDF) [file pone.0133869.s002.pdf]

WHO Region and Country		Study Design	Study Setting	Cohort Type	Sample Size	Specimen Type	Phenotypic Method	PZase Activity Assay	Genotypic Method	Reference
Africa	**Central African Republic**	Retrospective	Lab	N/A	54	Sputum	N/A	No	PCR and DNA sequencing	Minime-Lingoupou 2010
	**Congo**	Retrospective	Lab	MDR	1	MTB clinical isolates	BACTEC 460 (100µg/ml)	Yes	PCR and DNA sequencing	Mestagh 1999
	**Kenya**	Prospective	Clinical	Any TB	286	MTB clinical isolates	BACTEC 960 (100µg/ml)	No	N/A	Ndung'u 2012
	**Rwanda**	Retrospective	Lab	MDR	1	MTB clinical isolates	BACTEC 460 (100µg/ml)	Yes	PCR and DNA sequencing	Mestagh 1999
	**Tanzania**	Retrospective	Lab	N/A	13	MTB clinical isolates	N/A	No	PCR and DNA sequencing	Mpagama 2013
	**South Africa**	N/S	N/S	HR-MDR	27	MTB clinical isolates	BACTEC 460 (100µg/ml)	Yes	PCR and DNA sequencing	Davies 2000
		Retrospective	Lab	N/A	9	MTB clinical isolates	N/A	No	Whole genome sequencing	Ioerger 2009
		Retrospective	Lab	Any TB	26	MTB clinical isolates	BACTEC 960 (100µg/ml)	No	Ion Torrent	Daum 2012
		Retrospective	Lab	N/A	21	MTB clinical isolates	BACTEC 460 (100-900µg/ml)	Yes	PCR and DNA sequencing	Bishop 2001
		Retrospective	Clinical	HR-MDR	174	MTB clinical isolates	BACTEC 960 (100µg/ml)	No	PCR and DNA sequencing	Louw 2006
		Retrospective	Lab	MDR; any TB	221	MTB clinical isolates	BACTEC 460 (100µg/ml)	Yes	PCR and DNA sequencing	Mphahlele 2008
Americas	**Brazil**	Retrospective	Lab	N/A	40	MTB clinical isolates	Indirect proportion method	Yes	PCR and DNA sequencing	Rodrigues 2005
		Retrospective	Lab	N/A	36	MTB clinical isolates	Proportion method	Yes	PCR and DNA sequencing	Barco 2006
		Prospective	Clinical	HR-MDR	23	Sputum	BACTEC 460 (N/S)	Yes	PCR and DNA sequencing	Clemente 2008
		Prospective	Clinical	Any TB	71	MTB clinical isolates	7H9 agar	Yes	N/A	Ribeiro 2012
		Retrospective	Clinical	Any TB	97	MTB clinical isolates	Proportion method	Yes	PCR and DNA sequencing	Bhuju 2013
		Prospective	Lab	Any TB	50	Sputum	BACTEC 460 (100µg/ml)	No	N/A	Barreto 2002
		Retrospective	Lab	N/A	32	MTB clinical isolates	N/A	Yes	PCR and DNA sequencing	Ghiraldi 2011
	**Canada**	Retrospective	Lab	Any TB	28	MTB clinical isolates	Middlebrook 7H11	Yes	PCR and DNA sequencing	Hirano 1998
		Retrospective	Lab	N/A	28	MTB clinical isolates	BACTEC 460 (100-300µg/ml)	Yes	PCR and DNA sequencing	Cheng 2000
		Prospective	Lab	Any TB	743	MTB clinical isolates	BACTEC 460/960 (100µg/ml)	Yes	PCR and DNA sequencing	Chedore 2010
		Retrospective	Lab	Any TB	141	MTB clinical isolates	BACTEC 460 (100µg/ml)	No	PCR and DNA sequencing	Alexander 2012
		Retrospective	Lab	MDR	1	MTB clinical isolates	BACTEC 460 (100µg/ml)	Yes	PCR and DNA sequencing	Mestagh 1999
		Retrospective	Lab	N/A	354	MTB clinical isolates	BACTEC 460 (100µg/ml)	Yes	PCR and RFLP	Nguyen 2003
	**Cuba**	Retrospective	Lab	Any TB	102	MTB clinical isolates	Nitrate reductase assay	Yes	PCR and DNA sequencing	Mirabal 2010
	**Mexico**	Prospective	Lab	HR-MDR	127	Sputum	BACTEC 960 (100µg/ml)	No	PCR and DNA sequencing	Cuevas-Cordoba 2013
		Prospective	Lab	Any TB	460	MTB clinical isolates	BACTEC 460 (N/S)	No	N/A	Granich 2000
	**Peru**	Prospective	Clinical	HR-MDR	29	MTB clinical isolates	N/A	Yes	PCR and DNA sequencing	Escalante 1998
		Retrospective	Lab	Any TB	185	MTB clinical isolates	BACTEC 460 (100µg/ml)	Yes	PCR and DNA sequencing	Sheen 2009
		Prospective	Clinical	MDR	150	MTB clinical isolates	BACTEC 460 (100µg/ml)	No	N/A	Becerra 2000
		Retrospective	Clinical	HR-MDR	122	MTB clinical isolates	N/A	Yes	N/A	Saravia 2005
	**United States**	Retrospective	Lab	Any TB	16	MTB clinical isolates	BACTEC 460 (N/S)	No	PCR and DNA sequencing	Sachais 1998
		Retrospective	Lab	MDR; Any TB	48	MTB clinical isolates	BACTEC 460 (100µg/ml)	No	N/A	LaBombardi 2002
		Retrospective	Lab	N/A	19	MTB clinical isolates	BACTEC 460 (100-900µg/ml)	Yes	PCR and DNA sequencing	Cheng 2000
		Retrospective	Lab	N/A	60	MTB clinical isolates	Middlebrook 7H11	Yes	PCR and DNA sequencing	Morlock 2000
		Retrospective	Lab	N/A	1436	MTB clinical isolates	BACTEC 460 (100µg/ml)	No	PCR and DNA sequencing	Hannan 2001
		Retrospective	Lab	N/A	33	MTB clinical isolates	BACTEC 460 (100µg/ml)	Yes	PCR and DNA sequencing	Denkin 2005
		Retrospective	Lab	Any TB	54	MTB clinical isolates	BACTEC 460/960 (100µg/ml)	No	N/A	Fredriks 2006
		Retrospective	Lab	N/A	29	MTB clinical isolates		No	PCR and DNA sequencing	Dormandy 2007

		Prospective	Lab	Any TB	27	MTB clinical isolates	BACTEC 460 (6.25-50µg/ml)<br>BACTEC 960 (100µg/ml)	No	N/A	Duque 2013
**East Med.**	**Iran**	Prospective	Clinical	N/A	49	MTB clinical isolates	Middlebrook 7H9	Yes	PCR and DNA sequencing	Doustdar 2009
	**Yemen**	Retrospective	Lab	Any TB	31	MTB clinical isolates	Middlebrook 7H11	Yes	PCR and DNA sequencing	Hirano 1998
	**Pakistan**	Prospective	Clinical	MDR	30	MTB clinical isolates	BACTEC 960 (50-100µg/ml)	No	N/A	Khurram 2012
	**Saudi Arabia**	Prospective	Lab	Any TB	101	MTB clinical isolates	BACTEC (N/S)	No	N/A	Khan 2001
**Europe**	**Abkhazia**	Prospective	Clinical	MDR	37	Sputum	BACTEC 960 (100µg/ml)	No	N/A	Pardini 2007
	**Azerbaijan**	Retrospective	Lab	MDR	5	MTB clinical isolates	BACTEC 460 (100µg/ml)	Yes	PCR and DNA sequencing	Mestagh 1999
	**Belgium**	Retrospective	Lab	N/A	138	MTB clinical isolates	BACTEC 960 (100µg/ml)	No	PCR and DNA sequencing	Stoffels 2012
		Retrospective	Lab	MDR	2	MTB clinical isolates	BACTEC 460 (100µg/ml)	Yes	PCR and DNA sequencing	Mestagh 1999
	**France**	Retrospective	Lab	Any TB	35	MTB clinical isolates	Middlebrook 7H10	Yes	PCR and DNA sequencing	Lemaitre 1999
	**Germany**	Retrospective	Lab	N/A	9	MTB clinical isolates	BACTEC 460 (100µg/ml)	No	PCR and DNA sequencing	Napiorkowska 2014
	**Greece**	Prospective	Lab	Any TB	150	MTB clinical isolates	BACTEC 460/960 (100µg/ml)	No	N/A	Kontos 2003
	**Italy**	Retrospective	Lab	Any TB	337	MTB clinical isolates	BACTEC 460 (N/S)	No	N/A	Nutuni 1998
		Retrospective	Lab	MDR; HR-MDR	46	MTB clinical isolates	BACTEC 460 (100µg/ml)	No	N/A	Fattorini 1999
		Retrospective	Lab	Any TB	100	MTB clinical isolates	BACTEC 460/960 (100µg/ml)	No	N/A	Scarparo 2004
		N/S	N/S	MDR; Any TB	201	MTB clinical isolates	BACTEC 960 (100µg/ml)	No	PCR and DNA sequencing	Piersimoni 2013
	**Latvia**	Retrospective	Clinical	N/A	28	MTB clinical isolates	BACTEC 460 (N/S)	No	PCR and DNA sequencing	Tracevska 2004
	**Netherlands**	Retrospective	Lab	Any TB	1831	MTB clinical isolates	BACTEC 960 (100µg/ml)	No	PCR and DNA sequencing	Simons 2012
	**Poland**	Retrospective	Lab	Any TB	33	MTB clinical isolates	BACTEC 960 (100µg/ml)	Yes	PCR and DNA sequencing	Sekiguchi 2007
		Retrospective	Lab	Any TB	1909	MTB clinical isolates	BACTEC 460 (100µg/ml)	No	N/A	Napiorkowska 2010
		Retrospective	Lab	N/A	72	MTB clinical isolates	BACTEC 460 (100µg/ml)	No	PCR and DNA sequencing	Napiorkowska 2014
	**Portugal**	Retrospective	Clinical	N/A	55	MTB clinical isolates	BACTEC 960 (100µg/ml)	Yes	PCR and DNA sequencing	Portugal 2004
		Retrospective	Lab	MDR	58	MTB clinical isolates	BACTEC 960 (100µg/ml)	No	PCR and DNA sequencing	Perdigao 2008
	**Russia**	Prospective	Clinical	N/A	44	MTB clinical isolates	BACTEC 460 (100µg/ml)	Yes	PCR and DNA sequencing	Marttila 1999
		Prospective	Prison	N/A	75	MTB clinical isolates	N/A	No	PCR and DNA sequencing	Shemyakin 2004
		Prospective	Clinical	N/A	21	MTB clinical isolates	N/A	No	PCR and DNA sequencing	Zhdanova 2013
		Retrospective	Lab	MDR	3	MTB clinical isolates	BACTEC 460 (100µg/ml)	Yes	PCR and DNA sequencing	Mestagh 1999
	**Spain**	Retrospective	Lab	N/A	50	MTB clinical isolates	BACTEC 460 (100µg/ml)	No	PCR and DNA sequencing	Aragon 2007
		Retrospective	Clinical	Any TB	57	MTB clinical isolates	BACTEC 460 (100µg/ml)	No	PCR and DNA sequencing	Espasa 2012
	**Scotland**	Retrospective	Lab	Any TB	6	MTB clinical isolates	BACTEC 460 (100µg/ml)	Yes	PCR and DNA sequencing	Mestagh 1999
	**Sweden**	Retrospective	Lab	N/A	69	MTB clinical isolates	BACTEC 460 (100µg/ml)	No	PCR and DNA sequencing	Jureen 2008
		Prospective	Lab	N/A	52	MTB clinical isolates	BACTEC 960 (100µg/ml)	No	PCR and DNA sequencing	Hoffner 2013
		Retrospective	Clinical	N/A	46	MTB clinical isolates	BACTEC 460/960 (100µg/ml)	Yes	PCR and DNA sequencing	Werngren 2012
	**Turkey**	Retrospective	Lab	N/A	10	MTB clinical isolates	BACTEC 460 (100µg/ml)	Yes	PCR and DNA sequencing	Brown 2000
		Retrospective	Lab	N/A	12	MTB clinical isolates	BACTEC 460 (N/S)	Yes	PCR and DNA sequencing	Yuksel 2009
	**United Kingdom**	Retrospective	Lab	Any TB	20899	MTB clinical isolates	BACTEC 460 (100µg/ml)	No	N/A	Djuretic 2002
**South East Asia**	**Bangladesh**	Retrospective	Lab	MDR	4	MTB clinical isolates	BACTEC 460 (100µg/ml)	Yes	PCR and DNA sequencing	Mestagh 1999
	**India**	Retrospective	Lab	Any TB	10	MTB clinical isolates	Middlebrook 7H11	Yes	PCR and DNA sequencing	Hirano 1998
		Prospective	Clinical	N/A	65	Sputum	N/A	No	PCR and DNA sequencing	Stavrum 2009
		Retrospective	Clinical	N/A	77	MTB clinical isolates	N/S	No	PCR and DNA sequencing	Harris 2000
		Prospective	Clinical	HR-MDR	50	Sputum	Proportion method	Yes	PCR and DNA sequencing	Muthaiah 2010

		N/S	Clinical	Any TB	130	MTB clinical isolates	BACTEC 960 (100µg/ml)	Yes	N/A	Krishnamurthy 2004
		Retrospective	Lab	N/A	107	MTB clinical isolates	Proportion method	Yes	N/A	Singh 2007
		Prospective	Clinical	N/A	63	MTB clinical isolates	BACTEC 960 (100µg/ml)	Yes	PCR and DNA sequencing	Shenai 2009
		Retrospective	Lab	Any TB	136	MTB clinical isolates	BACTEC 960 (100µg/ml)	Yes	N/A	Sharma 2010
		Retrospective	Lab	Any TB	186	MTB clinical isolates	BACTEC 960 (100µg/ml)	No	N/A	Arora 2013
	**East Timor**	Prospective	Clinical	MDR; HR-MDR	23	MTB clinical isolates	N/A	Yes	N/A	Kelly 2004
	**Indonesia**	Retrospective	Lab	Any TB	8	MTB clinical isolates	Middlebrook 7H11	Yes	PCR and DNA sequencing	Hirano 1998
		Prospective	Clinical	Any TB	103	MTB clinical isolates	N/A	Yes	N/A	Kelly 2006
	**Myanmar**	Retrospective	Lab	Any TB	16	MTB clinical isolates	Middlebrook 7H11	Yes	PCR and DNA sequencing	Hirano 1998
	**Thailand**	Retrospective	Lab	Any TB	54	MTB clinical isolates	Middlebrook 7H11	Yes	PCR and DNA sequencing	Hirano 1998
		Prospective	Lab	Any TB	30	MTB clinical isolates	BACTEC 960 (100µg/ml)	No	N/A	Duque 2013
		Retrospective	Lab	N/A	42	MTB clinical isolates	BACTEC 960 (100µg/ml)	No	PCR and DNA sequencing	Watcharasamphankul 2013
		Retrospective	Lab	N/A	98	MTB clinical isolates	BACTEC 960 (100µg/ml)	No	PCR and DNA sequencing	Pholwat 2014
		Retrospective	Lab	MDR; Any TB	150	MTB clinical isolates	BACTEC 960 (100µg/ml)	Yes	PCR and DNA sequencing	Jonmalung 2010
**Western Pacific**	**Cambodia**	Retrospective	Lab	N/A	166	MTB clinical isolates	N/A	No	PCR and DNA sequencing	Pierre-Audigier 2012
	**China**	Retrospective	Lab	N/A	64	MTB clinical isolates	BACTEC 460 (100µg/ml)	No	PCR and DNA sequencing	Hou 2000
		Retrospective	Lab	N/A	47	MTB clinical isolates	Proportion method	Yes	PCR and DNA sequencing	Zhang 2009
		Prospective	Clinical	Any TB	51	MTB clinical isolates	BACTEC 960 (100µg/ml)	Yes	PCR and DNA sequencing	Zhou 2011
		Prospective	Clinical	N/A	53	Sputum	N/A	Yes	Long fragment qPCR	Li 2014
		Prospective	Clinical	Any TB	432	MTB clinical isolates	BACTEC 960 (100µg/ml)	Yes	PCR and DNA sequencing	Cui 2013
		Retrospective	Clinical	Any TB	132	MTB clinical isolates	Proportion method	Yes	PCR and DNA sequencing	Huang 2013
		Prospective	Clinical	MDR; Any TB	218	MTB clinical isolates	BACTEC 960 (100µg/ml)	No	PCR and DNA sequencing	Tan 2013
	**Malaysia**	Retrospective	Lab	Any TB	1	MTB clinical isolates	Middlebrook 7H11	Yes	PCR and DNA sequencing	Hirano 1998
	**New Zealand**	Prospective	Lab	N/A	33	MTB clinical isolates	BACTEC 460/960 (100µg/ml)	Yes	PCR and DNA sequencing	Pandey 2009
	**Philippines**	Retrospective	Lab	Any TB	12	MTB clinical isolates	Middlebrook 7H11	Yes	PCR and DNA sequencing	Hirano 1998
	**Papua New Guinea**	Retrospective	Clinical	MDR	39	MTB clinical isolates	BACTEC 460/960 (100µg/ml)	No	N/A	Simpson 2011
	**Korea**	Retrospective	Lab	Any TB	8	MTB clinical isolates	Middlebrook 7H11	Yes	PCR and DNA sequencing	Hirano 1998
		Retrospective	Lab	N/A	10	MTB clinical isolates	Proportion method	Yes	PCR and DNA sequencing	Cheng 2000
		Retrospective	Lab	N/A	95	MTB clinical isolates	N/A	Yes	PCR and DNA sequencing	Lee 2001
		Retrospective	Clinical	HR-MDR	93	Sputum	N/A	Yes	PCR and DNA sequencing	Choi 2010
		Retrospective	Lab	N/A	80	MTB clinical isolates	N/A	Yes	PCR and DNA sequencing	Yoon 2012
		Prospective	Clinical	N/A	102	MTB clinical isolates	Proportion method	Yes	PCR and DNA sequencing	Kim 2012
		Retrospective	Clinical	N/A	23	Extrapulmonary MTB	N/A	No	PCR and DNA sequencing	Lee 2012
		Retrospective	Lab	N/A	192	MTB clinical isolates	Absolute concentration method	No	PCR and DNA sequencing	Jnawali 2013
	**Taiwan**	Retrospective	Clinical	MDR; Any TB	76	MTB clinical isolates	BACTEC 960 (100-300µg/ml)	Yes	PCR and DNA sequencing	Huang 2003
		Retrospective	Clinical	Any TB	611	MTB clinical isolates	N/A	Yes	N/A	Su 2008
		Prospective	Clinical	MDR	66	MTB clinical isolates	BACTEC 960 (100µg/ml)	Yes	PCR and DNA sequencing	Chiu 2011
		Prospective	Lab	MDR; Any TB	56	MTB clinical isolates	BACTEC 960 (100µg/ml)	No	N/A	Huang 2013
	**Vietnam**	Retrospective	Clinical	Any TB	339	MTB clinical isolates	N/A	Yes	Line probe	Van Hung 2013
