# Supplementary material for: A Global Perspective on Pyrazinamide Resistance: Systematic Review and Meta-Analysis
Source: PLoS One. 2015 Jul 28;10(7):e0133869. doi: 10.1371/journal.pone.0133869 (PMC4517823; doi:10.1371/journal.pone.0133869)
Supplement: S2 Table — Abbreviations: A, adenine; bp, base pair; C, cytosine; del, deletion; G, guanine; R, resistant; SNP, single-nucleotide polymorphism; T, thymine. * Article found one isolate sensitive and one isolate resistant. (PDF) [file pone.0133869.s003.pdf]

SNP location	SNP type	Country and reference	Phenotype linked to SNP	SNP Frequency
-71	1-bp deletion	Korea[1]	R	5
	2-bp deletion	Korea[1]	R	1
-71 & -11	1-bp deletion & A>G	Korea[1]	R	2
-71 & 20	1-bp deletion & T>G	Korea[1]	R	1
-71 & 56 & 386	1-bp deletion & A insertion & 10-bp insertion	Korea[1]	R	1
-71 & 58 & 400	1-bp deletion & G>A & G>A	Korea[1]	R	1
-71 & 67	1-bp deletion & C insertion	Korea[1]	R	1
-71 & 152	1-bp deletion & A>C	Korea[1]	R	1
-71 & 161	2-bp deletion & C>T	Korea[1]	R	1
-71 & 199	1-bp deletion & T>C	Korea[1]	R	1
-71 & 226	1-bp deletion & A>C	Korea[1]	R	1
-71 & 298	1-bp deletion & A>C	Korea[1]	R	1
-71 & 307	1-bp deletion & T>C	Korea[1]	R	1
-71 & 403	1-bp deletion & A>C	Korea[1]	R	3
-71 & 446 & 501	1-bp deletion & T insertion and T insertion	Korea[1]	R	1
-71 & 454	1-bp deletion & 10-bp deletion	Korea[1]	R	1
-71 & 478	1-bp deletion & A>C	Korea[1]	R	1
-16	AACGTA>GGCAGTT	USA[33]	R	1
-15 & 11	A>C & T>C	Sweden[57]	R	1
-13	G>T	Canada[64]	R	1
-12	T>C	South Africa[3], Netherlands[41]	R[3,41]	3
	T>G	Russia[4]	R	1
	T deletion	Iran[2]	R	1
-12 & 228	T>C & T>C	Korea[5]	R	1
-11	80-bp deletion	Korea[1]	R	1
-11 & 376	80-bp deletion & G>A	Korea[1]	R	1
-11	A>G	Korea[1,5,6,7], Iran[2], Russia[4], Thailand[8], Portugal[9], Scotland[10], Brazil[49], Canada[12,64], Peru[13], China[31,47], Sweden[38], India[50], Spain[59], Poland[66]	R[1,2,4,5,6,7,8,9,10,12,13,31,38,47,49,50,59,64,66]	62
-11 & 483	A>G & G>A	India[50]	R	1
-11 & 535	A>G & A deletion	India[50]	R	1
-11	A>C	Thailand[8]	R	1
-10	T>C	South Africa[3]	R	1
-7	T>C	USA[33]	R	1
-7 & 340	T>C & A>G	Japan[14]	R	2
-3	C insertion	Sweden[57]	*R/S	1
1	561-bp deletion	Japan[46]	R	1
	11-bp deletion	China[15]	R	1
	8-bp deletion	China[15]	R	1
2	T>C	Portugal[9,35], Poland[66]	R[9,35,66]	9
3	G>A	Japan[16], Netherlands[41]	R[16,41]	4
	G>T	Korea[6]	R	1
7	G>C	Iran[2], Brazil[11,17], France[37]	R[2,11,17,37]	4
	G>T	Taiwan[18]	R	1
8	C>A	Korea[1, 20], Japan[14,19,46]	R[1,14,19,20,46]	6
8 & 419	C>A & G>A	Japan[14]	R	1
11	T>C	Korea[1], Peru[32], USA[33], China[34,47], Japan[36]	R[1,32,33,34,36,47]	7
	T>G	Korea[1,6]	R[1,6]	2
12	G deletion	Cuba[56]	R	1
12 & 421	T insertion & C>T	Korea[1]	R	1
13	AT insertion	India[58]	R	2
14	T>A	Japan[16,46]	R[16,46]	2
	T>G	Japan[16], India[50]	R[16,50]	3
16	T>C	South Africa[21]	R	1
17	T>G	Korea[20], China[34]	R[20,34]	2
	T>C	Sweden[38,57,61]	R[38,57,61]	3
19	G>A	Peru[13]	R	1
	G>T	Korea[6], Peru[13]	R[6,13]	2
19 & 22	G>C & G>T	Korea[1]	R	1
19 & 403	G>C & A>C	Korea[1]	R	1

20	T>G	Russia[4], Yemen[22], Brazil[23], USA[39]	R[4,22,23,39]	5
	T>A	Korea[6]	R	1
	T>C	Brazil[11]	R	1
20 & 386 & 517	C insertion & 10-bp insertion & G deletion	Korea[1]	R	1
20 & 437	C insertion & C>T	Korea[1]	R	1
22	G>T	Russia[4], Belgium[43]	R[4,43]	2
	G>A	South Africa[3], China[34], Belgium[43]	R[3,34,43]	4
22 & 104	G>T & T>G	South Africa[3]	R	1
23	A>G	Korea[6,20], Spain[59], Canada[64]	R[6,20,59,64]	5
	A>C	China[31]	R	1
	TCG insertion	Korea[7]	R	1
24	C>G	Japan[46]	R	1
25	G>T	South Africa[24]	R	1
26	T>G	Korea[6], Japan[14]	R[6,14]	2
	T>C	Korea[6]	R	1
28	C>T	Russia[4], Korea[6,20], Brazil[11], Latvia[25], Belgium[43]	R[4,6,11,20,25,43]	7
	C>A	Russia[4], Spain[59]	R[4,59]	2
	C deletion	China[15]	R	2
29	A>C	Korea[1,6,20], Myanmar[22], China[31,48], USA[33,39], Japan[36], Belgium[43], Spain[59], Singapore[62], Poland [19,66]	R[1,6,19,20,22,31,33,36,39,43,48,59,62,66]	17
	A>G	Korea[6], USA[12], Peru[32], Cuba[56], Poland[66]	R[6,12,32,56,66]	7
29 & 295	A>C & T>G	Korea[6]	R	1
31 & 34 & 290	C insertion & G>T & G>A	Korea[1]	R	1
34	G>A	Korea[5], USA[39], Italy[40], China[63]	R[5,39,40,63]	7
35	A>C	Korea[6,20] ,USA[12], China[15,47], Japan[19], India[58], Mexico[26], Peru[32], Canada[64]	R[6,12,15,19,20,26,32,47,58,64]	20
	A>G	South Africa[3]	*R/S	9
	G insertion	South Africa[3]	R	2
36	A>G	Peru[32]	R	2
37	T>C	China[31]	R	1
38	T>C	France[37]	R	1
40	T>C	Korea[1,5,6], South Africa[3,24,60], Myanmar[22], Japan[46], China[47]	R[1,3,5,6,22,24,46,47,60]	14
	T>G	Peru[32]	R	3
	T deletion	South Africa[3]	R	1
41	G>A	Korea[6,7], Latvia[25], China[34]	R[6,7,25,34]	4
42	C>G	Korea[20]	R	1
	C>A	Poland[66]	R	1
42 & 307	C>G & T>C	Latvia[25]	R	1
42 & 535	C>A & A deletion	India[50]	R	1
46	G>A	China[47]	R	1
49	G>A	China[34], Singapore[62]	R[34,62]	2
	1-bp deletion	Korea[20]	R	1
50	G>A	Thailand[22]	R	1
52	T>C	New Zealand[45]	R	1
	G insertion	Canada[12]	R	1
52 &161	T>C & C>T	China[31]	R	1
54	2-bp insertion	Japan[16]	R	1
56	T>C	China[15], New Zealand[45]	R[15,45]	2
	T>G	Thailand[8]	R	1
	234-bp deletion	Korea[7]	R	1
57 & 62 & 70	3-bp deletion & 4-bp deletion & 5-bp deletion	China[48]	R	1
59	1-bp deletion	China[15]	R	1
59 & 232	1-bp deletion & G>A	Japan[14]	R	1
60	G deletion	Netherlands[41]	R	1
61	G insertion	Italy[40], China[47]	R[40,47]	2
64	A deletion	Thailand[65]	R	1
67	1-bp deletion	Korea[20]	R	1
	C insertion	Korea[1]	R	1
68	G>C	Taiwan[18]	R	1
68-69	GT>TC	Korea[6]	R	1
71	G>A	Turkey[27], China[31], Peru[32]	R[27,31,32]	5

	G deletion	China[15], USA[33,39]	R[15,33,39]	5
74	1-bp deletion	Korea[20]	R	1
	5-bp deletion	Brazil[17]	R	1
74-75 & 77 & 83	CC>AA & C>G & C>A	Korea[6]	R	1
76	GC insertion	Brazil[17]	R	2
77	5-bp insertion	Korea[1]	R	1
	G deletion	Korea[6]	R	1
78	G>C	India[58]	R	2
80	T>C	Thailand[8], China[47], India[50]	R[8,47,50]	4
83	C>T	Taiwan[18]	R	1
84	C deletion	Russia[4]	R	1
89	C>A	China[51]	R	1
92	T>C	Thailand[8]	*R/S	13
	T>G	Thailand[8]	*R/S	4
94	T>G	South Africa[24]	R	1
96 & 338	C>T & G>T	Russia[42]	R	3
96 & 338 & 362	C>T & G>T & G>T	Russia[42]	R	1
98	A>C	China[34]	R	1
99	C deletion	Sweden[38]	R	1
100	T>G	Japan[14,16,46], Peru[32]	R[14,16,32,46]	6
100 & 400	T>G & G>T	Japan[14]	R	1
101	A>G	Iran[2]	R	1
	A>C	Thailand[22]	R	2
102	C>A	Korea[5], Japan[14]	R[5,14]	2
	C>G	Netherlands[41]	R	1
	167-bp deletion	Turkey[27]	R	3
103	C deletion	Spain[59]	R	1
104	T>C	Thailand[8], Japan[46], Spain[59]	R[8,46,59]	4
	T>G	Brazil[23]	R	1
106	~5.3 kb deletion	USA[33]	R	1
107	CG insertion	South Africa[21]	R	1
108	C>G	China[47]	R	1
109	G>T	South Africa[24]	R	3
117 & 169	G>A & C>G	Sweden[38]	R	1
121	T>C	China[15]	R	1
123	C>G	China[15]	R	2
	C>A	Thailand[22], Peru[32]	R[22,32]	3
128	A>C	Thailand[22], Belgium[43]	R[22,43]	3
129 & 493	2-bp deletion & G>A	Japan[14]	R	1
132	A insertion	Korea[1]	R	1
134	T>A	Korea[1]	R	2
	T>G	Korea[6], Taiwan[18]	R[6,18]	2
	T>C	Thailand[8], China[34]	R[8,34]	2
135	G>C	Taiwan[18]	R	1
136	G>C	South Africa[24], Spain[59]	R[24,59]	2
	G>T	Thailand[8]	R	1
	G insertion	Yemen[22]	R	1
	G deletion	Brazil[17]	R	1
137	C>A	Korea[1,6,12]	R[1,6,12]	3
	C>T	Portugal[9], Canada[12,64], USA[12], Korea[12], Yemen[22]	R[9,12,22,64]	9
139	A>C	China[15], Korea[20]	R[15,20]	2
	A>G	Korea[6], Mexico[26], China[31], USA[33,39], Sweden[38]	R[6,26,31,38,39], *R/S[33]	12
	A>T	Russia[4]	R	1
142	A>G	Korea[1], China[47]	R[1,47]	3
143	A>C	Peru[32], Cuba[56]	R[32,56]	5
145	G>A	Korea[20], Peru[32]	R[20,32]	28
	G>C	South Africa[24]	R	1
	G>T	China[48]	R	1
145 & 305	G>C & C>T	Mexico[26]	R	1
146	A>T	Russia[4]	R	1
	A>G	Russia[4], Korea[20]	R[4,20]	2
	A>C	Korea[6,20], Brazil[23], Belgium[43], Japan[46]	R[6,20,43,46], *R/S[23]	8

150	80-bp deletion	Korea[20]	R	1
151	C>G	China[51]	R	1
	C>A	Brazil[17]	R	1
	C>T	Scotland[10]	R	1
	80-bp deletion	Korea[6]	R	1
152	A>C	Portugal[9], Korea[20], Thailand[22], Turkey[27], USA[33], Japan[46], China[47]	R[9,20,22,27,33,46,47]	10
	A>G	Brazil[11], Thailand[22], Peru[32], Italy[40], India[50], Canada[64]	R[11,22,32,40,50,64]	21
153	C>A	Japan[19], Sweden[38], USA[39], Poland[66]	R[19,38,39,66]	6
	C>G	Poland[19,66], India[58], Sweden[61]	R[19,58,61,66]	7
153 & 493	C>A & G>A	Japan[14]	R	1
156 & 546	C>A & G>T	India[50]	R	1
157	G>A	Japan[19]	R	1
158	A>C	Korea[12]	R	1
	44-bp deletion	China[47]	R	1
	A deletion	Korea[6]	R	1
160	C>A	China[15]	R	1
	C>T	Sweden[38,61]	R[38,61]	2
161	C>G	Japan[16], Canada[64]	R[16,64]	2
	C>T	Japan[14,19], Taiwan[18], South Africa[21,24], China[34,47], Peru[32], Sweden[38,61], Spain[59]	R[14,18,19,21,24,32,34,38,47,59,61]	15
	C>A	Brazil[11], Korea[20], China[34]	R[11,20,34]	3
	C deletion	Korea[6]	R	1
161 & 225	C>T & T>C	Mexico[26]	R	1
165	T>A	Taiwan[18]	R	1
166	G>T	Taiwan[18]	R	1
167	A>G	Taiwan[18]	R	1
168	C insertion	Taiwan[18]	R	1
169	C>G	Iran[2], Korea[6], Sweden[38,57], USA[39,55], New Zealand[45], Japan[46], Canada[64]	R[2,6,38,39,45,46,55,57,64]	21
	C>T	South Africa[24], China[31,48], Netherlands[41]	R[24,31,41,48]	7
169 & 222	C>G & C>T	Canada[64]	R	1
170	A>C	Taiwan[18], China[34], Turkey[44], Japan[46]	R[18,34,44,46]	4
	A>G	Korea[5], Brazil[11], Canada[64]	R[5,11,64]	3
	T insertion	China[34]	R	1
171	C>T	Taiwan[18]	R	1
172	T>C	Korea[6,7], Peru[13], Sweden[38,61]	R[6,7,13,38,61]	6
	T deletion	South Africa[24]	R	12
	2-bp deletion	Brazil[23]	R	1
173	T>C	China[31,47]	R[31,47]	2
174	C>A	Taiwan[18], South Africa[30]	R[18,30]	2
	C>G	Korea[20], Canada[22,64], Sweden[38]	R[20,22,38,64]	4
	T insertion	China[34]	R	1
175	T>C	South Africa[3], Brazil[11,49], Japan[14], Korea[20], Turkey[44]	R[3,11,14,20,44,49]	6
180 & 425	C>T & C>T	Korea[7]	R	1
181	A>C	France[37]	R	1
181 & 416	A>C & T>C	Japan[46]	R	1
182	5-bp insertion	Brazil[17]	R	3
	CA deletion	Japan[46]	R	1
184	C>A	Latvia[25], New Zealand[45], China[47]	R[25,45,47]	3
184 & 290	T insertion & G>T	Japan[46]	R	1
185	C>T	Japan[16], Korea[20], Peru[32], China[63]	R[16,20,32,63]	5
	C>A	Azerbaijan[10], Taiwan[18], Poland[66]	R[10,18,66]	3
	C>G	USA[33]	R	1
188	A>G	Yemen[22], Latvia[25], Belgium[43], Canada[64]	R[22,25,43,64]	5
	A>C	Mexico[26], Belgium[43],	R[26,43,62]	3

		Singapore[62]		
	2-bp insertion	Japan[16], Peru[32]	R[16,32]	2
	T insertion	New Zealand[45]	R	1
190	T>G	Korea[6,7]	R[6,7]	2
192	8-bp insertion	Brazil[23]	R	3
	A insertion	France[37], China[48]	R[37,48]	2
193	A insertion	Portugal[9], Canada[12], USA[12]	R[9, 12]	3
194 & 244	C>G & C>T	Turkey[44]	R	1
195	C>T	Iran[2], India[50], USA[39], Sweden[61]	R[2,39], *R/S[50,61]	19
	68-bp deletion	Azerbaijan[10]	R	1
195 & -11	C>T & A>G	India[50]	R	1
195 & 11	C>T & T>G	Canada[64]	R	1
195 & 29	C>T & A>G	India[50]	R	1
195 & 43	C>T & T deletion	Iran[2]	R	1
195 & 131	C>T & T>G	Iran[2]	R	1
195 & 182 & 226	C>T & A insertion & A deletion	Korea[1]	R	1
195 & 202	C>T & T>C	USA[39]	R	1
195 & 282	C>T & 2-bp deletion	Canada[64]	R	1
195 & 314	C>T & G>A	India[50]	R	1
195 & 328	C>T & G>T	India[50]	R	1
195 & 329	C>T & A deletion	Thailand[65]	R	1
195 & 391	C>T & G insertion	Canada[64]	R	1
195 & 406	C>T & G>A	India[50]	R	1
195 & 408	C>T & G insertion	Canada[64]	R	1
195 & 422	C>T & A>C	Canada[64]	R	1
195 & 515	C>T & T>C	India[50]	R	1
195 & 528	C>T & GC insertion	Sweden[38]	R	1
195 & 535	C>T & A>C	Iran[2]	R	1
195 & 535	C>T & A deletion	India[50]	R	1
196	T>C	Korea[1], Russia[4], China[31]	R[1,4,31]	3
	14-bp insertion	Korea[20]	R	1
199	T>C	Korea[6], Thailand[8], Canada[22], Sweden[38], China[47,48], Canada[64]	R[6,8,22,38,47,48,64]	7
200	8-bp insertion	India[50]	R	1
200 & 390	C>T & GG insertion	Thailand[65]	R	1
202	T>C	Korea[1,6], Brazil[17], South Africa[24], Sweden[38], China[47,48]	R[1,6,17,24,38,47,48]	11
	T>G	Korea[6], South Africa[24], Mexico[26], Poland[66]	R[6,24,26,66]	10
203	G>T	Brazil[11], Hong Kong[28], Spain[59]	R[11,28,59]	5
	G>C	Korea[1,6]	R[1,6]	2
	G>A	Korea[6,20], USA[12]	R[6,12,20]	4
205	C>G	Brazil[23]	R	1
206	C>G	Korea[20], India[58]	R[20,58]	4
	C>T	France[37]	R	1
207	ACC deletion	Taiwan[18]	R	1
211	C>T	South Africa[3,24], Taiwan[18], Mexico[26], Peru[32], Belgium[43], Canada[64]	R[3,18,24,26,32,43,64]	8
	C>G	Thailand[8]	R	8
	C>A	China[34], Belgium[43]	R[34,43]	2
	3-bp deletion	Korea[20]	R	1
212	A>G	Iran[2], Korea[7], Portugal[9], USA[33]	R[2,7,9,33]	6
	A>C	Japan[46]	R	1
	14-bp insertion	Korea[20]	R	1

213	T>A	Korea[1], Netherlands[41]	R[1,41]	3
	T>G	USA[12]	R	1
	T deletion	Brazil[17]	R	1
214	T>C	China[15], Thailand[22], Brazil[23], India[58]	R[15,22,23,58]	6
215	G>A	Thailand[8], Brazil[23], South Africa[24]	R[8,23,24]	3
	29-bp deletion	France[37]	R	1
216	C>A	Korea[20], China[47]	R[20,47]	2
	C>G	Japan[19], Latvia[25], Canada[64]	R[19,25,64]	4
218	G insertion	Netherlands[41]	R	1
221	G insertion	Scotland[10]	R	2
222	G>C	Thailand[8]	R	1
223	T>C	South Africa[21]	R	1
225 & 515	T>C & T>C	Mexico[26]	R	1
226	A>C	Korea[1,6,20], Brazil[17,23], South Africa[21], Myanmar[22], Latvia[25], Mexico[26], China[31,34,47,63], Peru[32], USA[33], Portugal[35], Netherlands[41], India[50], Cuba[56], Spain[59], Canada[64]	R[1,6,17,20,21,22,23,25,26,31,32,33,34,35,41,47,50,56,59,63,64]	38
	A>G	Taiwan[18]	R	1
227	C>T	Korea[6,7], Brazil[23], Turkey[44]	R[6,7,23,44]	4
232	G>A	Korea[5]	R	1
	G>T	Peru[32]	R	1
233	G>T	Taiwan[18]	R	1
	G>A	Korea[6], USA[33]	R[6,33]	2
	G insertion	USA[39]	R	1
233-237	GCGCG-ACGTCTCCA substitution	Korea[20]	R	1
234	G insertion	China[34]	R	1
	4-bp insertion	Korea[5]	R	1
236 & 360	C>T & T insertion	South Africa[3]	R	1
239	A deletion	Turkey[44]	R	1
241	T>G	China[31,63]	R[31,63]	3
244	C>G	Belgium[43]	R	1
245	A>G	USA[12], Brazil[17], China[34,63], Belgium[43], New Zealand[45]	R[12,17,43,45,63], *R/S[34]	7
	AT>GG	China[31]	R	1
245 & 389	A>G & T>C	Korea[20]	R	1
246	A>T	Brazil[17]	R	1
248	C>G	Taiwan[18]	R	1
249	T>G	China[34]	R	1
250	C insertion	Portugal[9,35]	R[9,35]	7
254	T>C	Iran[2], Korea[6], China[15], Taiwan[18], Peru[32], USA[33,39], India[58]	R[2,6,15,18,32,33,39,58]	15
	T>G	Korea[6,12], Azerbaijan[10], China[15], Japan[16], Latvia[25]	R[6,10,12,15,16,25]	7
	T>A	Canada[64]	R	2
258	18-bp insertion	Peru[32]	R	1
260	C>T	Belgium[43]	R	2
	C deletion	Canada[64]	R	1
	AC insertion	Japan[46]	R	1
260 & 436	C>T & G deletion	France[37]	R	1
261	2-bp insertion	Japan[14,16]	R[14,16]	4
263	C>A	China[15], Canada[64]	R[15,64]	2
264	24-bp deletion	USA[55]	R	1
269	T>G	South Africa[24], Sweden[38], USA[55], Canada[64], Thailand[65]	R[24,38,55,64,65]	5
279	106-bp insertion	Peru[32]	R	1
280	T>C	China[31], Peru[32]	R[31,32]	4
281	T>C	Mexico[26], China[31]	R[26,31]	2
	T>G	Cuba[56]	R	1
282	C>G	Korea[1] , Thailand[65]	R[1,65]	2
	T insertion	Portugal[35]	R	1
285	C>A	Portugal[35]	R	1

	CA deletion	Peru[32]	R	2
286	A>G	Russia[4], Korea[6,20], Portugal[9], China[31], India[50]	R[4,6,9,20,31,50]	14
	A>C	Korea[6]	R	1
	A>T	South Africa[3]	R	1
	A deletion	Korea[20]	R	2
287	A>C	Brazil[11,49], Korea[20], China[34], France[37]	R[11,20,34,3749]	7
	A>G	Mexico[26]	R	1
	A>T	Italy[40]	R	1
	T insertion	Korea[6], Canada[22]	R[6,22]	2
288	G>T	China[15]	R	2
	G>C	India[58]	R	37
	G deletion	USA[39]	R	1
289	G>T	South Africa[3,21], Italy[40], India[50]	R[3,21,40,50]	6
	G>A	Thailand[8], USA[12,33], Korea[20], China[34]	R[8,12,20,33,34]	8
	G>C	Netherlands[41]	R	1
290	G>A	South Africa[3], Japan[16], Taiwan[18,29], USA[33], China[34], New Zealand[45], Singapore[62]	R[3,16,18,29,33,34,45,62]	8
	T insertion	Portugal[9]	R	1
291	T>C	Sweden[57]	R	2
	T deletion	Italy[40]	R	1
292	G insertion	Poland[66]	R	1
293	10-bp deletion	Peru[32]	R	1
297	C>G	Brazil[17], USA[33]	R[17,33]	2
	C>A	Thailand[22], Japan[46]	R[22,46]	2
298	A>C	China[34]	R	4
	A>G	Taiwan[18]	R	1
300 & 539	C deletion & T>C	Singapore[62]	R	1
301	G deletion	Korea[12]	R	1
	A insertion	China[15]	R	1
303	A deletion	Korea[20]	R	1
304	G>A	Russia[4]	R	1
305	C>T	South Africa[3,60], Russia[4], China[34]	R[3,4,34,60]	8
306	C insertion	France[37]	R	1
307	T>G	Canada[64]	R	1
	T>C	Korea[6], Canada[10], Latvia[25], Sweden[38]	R[6,10,25,38]	10
	5-bp deletion	Korea[6]	R	1
308	A>T	Latvia[25]	R	1
	A>G	Taiwan[18], China[34], France[37], Poland[66]	R[18,34,37,66]	4
	A>C	Korea[6]	R	2
309	C>G	Russia[4], Korea[6], Brazil[17], South Africa[24], Peru[32], USA[33]	R[4,6,17,24,32,33]	15
	C>A	Korea[1], USA[33], Cuba[56]	R[1,33,56]	4
310	A>T	USA[55]	R	1
312	C>A	Korea[1,20]	R[1,20]	2
	C>G	Thailand[8,65], Yemen[22]	R[8,22,65]	4
314	G>A	Brazil[11], Peru[32], China[34]	R[11,32,34]	4
	C insertion	Brazil[11]	R	2
315	G insertion	South Africa[3], Brazil[17], China[34]	R[3,17,34]	4
316	245-bp deletion	Poland[66]	R	1
317	CT insertion	Korea[7]	R	1
319	G>A	South Africa[24]	R	1
	G>T	Taiwan[18]	R	1
322	G>T	Iran[2]	R	1
	G>C	Iran[2], Brazil[11], Korea[20]	R[2,11,20]	4
334	A>T	China[34]	R	1
335	A>T	Taiwan[18]	R	2
337	G deletion	Korea[20]	R	1
338 & 472	G>A & G>A	Korea[5]	R	1
339	G insertion	Canada[64]	R	1
340	A>C	Yemen[22], South Africa[24], Poland[66]	R[22,24,66]	4
341	C deletion	Korea[6,12]	R[6,12]	2
	1355-bp deletion	France[37]	R	1
346	C>G	South Africa[24]	R	1

347	T>G	Korea[1], Russia[4], India[50], Poland[66]	R[1,4,50,66]	11
	T>C	Peru[32], Italy[40], China[63]	R[32,40], *R/S[63]	4
350	T insertion	Japan[46]	R	1
352	7-bp deletion	Taiwan[29]	R	1
353	A>C	China[15]	R	1
355	T>A	China[34]	R	2
356	G>T	Peru[32]	R	1
357	G>A	Korea[6], Cuba[56]	R[6,56]	2
359	T>G	Iran[2], Brazil[11,49], New Zealand[45], India[50], Spain[59]	R[2,11,45,49,50,59]	6
	T>C	Portugal[9,35], Mexico[26], Sweden[57]	R[9,26,35,57]	29
	81-bp deletion	Peru[32]	R	2
359 & 435	T>G & G insertion	Korea[1]	R	1
361	C deletion	Spain[59]	R	1
362	G>C	Korea[6], China[15,48]	R[6,15,48]	3
	G>A	Thailand[8]	R	1
364	C>T	Taiwan[18], South Africa[21,60], Peru[32]	R[18,21,32,60]	4
368	G>C	Sweden[38,57,61]	R[38,57,61]	3
	G>T	South Africa[21]	R	1
	18-bp insertion	Korea[6]	R	1
	AG insertion	China[15]	R	1
373	G>T	Thailand[8], Sweden[38,61], New Zealand[45]	R[8,38,45,61]	4
	9-bp insertion	Korea[20]	R	1
374	T>G	Portugal[9,35], Mexico[26]	R[9,26,35]	28
	T>A	Russia[4], Sweden[38]	R[4,38]	3
	16-bp deletion	Japan[14]	R	1
375	C insertion	Mexico[26]	R	1
	9-bp deletion	Spain[59]	R	1
377	GA insertion	Peru[32]	R	1
378	10-bp deletion	China[47]	R	1
379	G>A	Iran[2]	R	1
	G>T	Thailand[8], China[34]	R[8,34]	2
	30-bp deletion	Mexico[26]	R	1
	11-bp deletion	Russia[4]	R	1
380	9-bp deletion	Mexico[26]	R	1
	11-bp deletion	Korea[20]	R	1
381	GG deletion	Korea[6]	R	1
	9-bp deletion	Portugal[35]	R	1
382	AG insertion	Korea[6,7]	R[6,7]	2
	8-bp deletion	Japan[36]	R	1
	9-bp deletion	India[50], Cuba[56]	R[50,56]	2
383	T>G	New Zealand[45], Japan[46], Sweden[57]	R[45,46,57]	3
	9-bp deletion	USA[39]	R	1
385	5-bp deletion	Korea[20]	R	1
	CG deletion	Japan[46]	R	1
385 & 420	T>G & GG insertion	USA[12]	R	1
386	T>G	Brazil[17]	R	1
	4-bp deletion	Korea[6]	R	1
388	9-bp insertion	Brazil[23], USA[33]	R[23,33]	2
389	T>C	Korea[1], China[34,47]	R[1,34,47]	4
	T>G	Canada[22], USA[33] , Thailand[65]	R[22,33,65]	4
	G insertion	India[50]	R	1
	GG insertion	Korea[20], South Africa[24], India[50]	R[20,24,50]	12
390	GG insertion	Mexico[26]	R	1
391	G>T	Korea[20]	R	1
	GG insertion	Korea[6], Portugal[9], France[37], Sweden[38,57], Japan[46], China[47]	R[6,9,37,38,46,47,57]	8
	G insertion	Mexico[26], China[47], Poland[66]	R[26,47,66]	3
392	G insertion	Korea[6], Portugal[35]	R[6,35]	2
	GG insertion	Canada[64]	R	1
393	GT insertion	Korea[7]	R	1
	T insertion	Korea[7]	R	1
	1-bp deletion	Korea[1]	R	1
394	G>A	South Africa[3], Taiwan[18], Japan[19], China[34], Sweden[38,61]	R[3,18,19,34,38,61]	7
395	G>T	Iran[2], Korea[7]	R[2,8]	3
	G>A	Portugal[9], Taiwan[18], Korea[20],	R[9,18,20,22,37,64]	7

		Myanmar[22], France[37], Canada[64]		
	G>C	China[31], Sweden[38,57,61], India[50]	R[31,38,50,57,61]	5
	9-bp insertion	Canada[64]	R	1
	17-bp deletion	India[22]	R	1
396	T insertion	China[48]	R	1
	TC insertion	India[58]	R	3
397	G insertion	Taiwan[29]	R	1
398	T>C	Russia[4], Japan[19], Portugal[35], China[63]	R[4,19,35,63]	5
	T>G	Brazil[11]	R	1
	T>A	USA[33]	R	1
	TT deletion	South Africa[21]	R	1
399	163-bp deletion	Singapore[62]	R	1
401	C>T	Canada[12], Peru[13], Taiwan[18,29], USA[33]	R[12,13,18,29,33]	5
403	A>C	Korea[1,6,7,20], China[31], Peru[32], USA[33], Japan[46], Poland[66]	R[1,6,7,20,31,32,33,46,66]	22
	A>G	Taiwan[18]	R	1
	CC insertion	China[15]	R	1
404	C>A	Poland[66]	R	3
406	G>A	Korea[6], Brazil[54]	R[6,54]	3
	G>C	Portugal[9], Scotland[10]	R[9,10]	2
	G>T	Korea[6]	R	1
	G deletion	Korea[6]	R	1
407	A>G	Korea[7,20], South Africa[24], China[31,63]	R[7,20,24,31], *R/S[63]	6
	C insertion	Azerbaijan[10]	R	1
	GC insertion	Japan[46]	R	1
408	A insertion	Korea[1], China[63], Canada[64]	R[1,63,64]	3
410	A>C	Korea[6], Bangladesh[10], Japan[14], Peru[32], China[47], India[58]	R[6,10,14,32,47,58]	7
	A>G	USA[33]	R	2
412	T>C	Japan[16,46]	R[16,46]	2
	G insertion	Thailand[8]	R	1
	T deletion	Japan[46]	R	1
413	G>A	China[15], Latvia[25], USA[33], Sweden[38], India[58]	R[15,25,33,38,58]	6
	G>C	Korea[12]	R	1
414	T>A	Korea[20]	R	2
	T>C	Brazil[17]	R	2
	G insertion	Korea[6]	R	1
415	G>C	China[15,31], Mexico[26], USA[33], India[58]	R[15,26,31,33,58]	6
	G>A	Korea[6,12], South Africa[24]	R[6,12,24]	6
	G>T	Japan[46]	R	1
415 & 418	G deletion & C>T	India[50]	R	1
416	T>C	China[15,31,47], Taiwan[18], Korea[20], Malaysia[22], South Africa[30], Japan[53], Cuba[56], Poland[66]	R[15,18,20,22,30,31,47,53,56,66]	13
	T>G	Thailand[8], China[31,34,48], USA[33], Portugal[35], Spain[59], South Africa[60], Singapore[62]	R[8,31,33,34,35,48,59,60,62]	13
418	G insertion	Korea[1,20]	R[1,20]	3
418 & 446	C>A & 8-bp deletion	Canada[12,64]	R	24
419	68-bp deletion	France[37]	R	1
420	G insertion	USA[12]	R	2
	GC insertion	South Africa[21]	R	1
421	C>T	Korea[6,7], Portugal[9]	R[6,7,9]	3
422	A>C	Korea[1,6,20], Russia[4], USA[12], China[31,47], India[58]	R[1,4,6,12,20,31,47,58]	10
	C deletion	Brazil[17]	R	1
424	A>G	Myanmar[22], China[34,47], Poland[66]	R[22,34,47,66]	4
425	C>A	China[15], Thailand[22], Sweden[38,61]	R[15,22,38,61]	7
	C>T	Thailand[8], Bangladesh[10],Korea[20], New Zealand[45], India[58]	R[8,10,20,45,58]	6
426	AG insertion	India[58]	R	1
427	G>A	Iran[2]	R	1
	G>C	Mexico[26]	R	1
428	C>G	Italy[40]	R	1
	GG insertion	Korea[12]	R	1

430	GAG deletion	Netherlands[41]	R	1
	6-bp deletion	Poland[66]	R	2
436	G>A	Korea[6,20], Thailand[8,52], China[63]	R[6,8,20,52,63]	7
437	C>T	India[22]	R	1
439	CG insertion	Portugal[9,35]	R[9,35]	4
440	1-bp deletion	Mexico[26]	R	1
442	C>A	Poland[19], Turkey[44]	R[19,44]	2
	C>T	Taiwan[18]	R	1
444	CG insertion	Brazil[23]	R	1
	12-bp insertion	Sweden[38,57]	R[38,57]	2
448	GC insertion	Korea[20]	R	2
449	G deletion	Japan[46]	R	1
451	T deletion	Korea[20]	R	1
452	T>C	South Africa[3], China[34,63], Canada[64]	R[3,34,63,65]	6
	T deletion	Korea[6]	R	1
454	G>A	Korea[5]	R	1
456	T insertion	Brazil[54]	R	2
457	C>G	Brazil[23]	R	1
458	C>A	USA[33]	R	1
	C>T	China[47]	R	2
458 & 474	C>T & C>A	China[48]	R	1
460	A>G	Korea[1], South Africa[3,60], Russia[4], China[34]	R[1,3,4,34,60]	5
461	G>C	Taiwan[18]	R	1
463	G>T	Korea[20]	R	1
	G>A	New Zealand[45], Canada[64]	R[45,64]	3
464	T>G	Iran[2], Korea[6,12], Brazil[11,17,49], Taiwan[29], France[37], Sweden[38,57,61], Belgium[43], Cuba[56]	R[2,6,11,12,17,29,37,38,43,49,56,57,61]	18
	T>C	Korea[20]	*R/S	1
	T insertion	Italy[40]	R	2
465	T insertion	Korea[6]	R	2
467	T>A	Japan[46]	R	1
470	T>G	Korea[1]	R	1
	T>C	Poland[66]	R	2
472	T>G	Thailand[65]	R	1
476	T>C	South Africa[3,24], Portugal[9], China[34]	R[3,9,24,34]	11
	T>G	Korea[6,20], China[31]	R[6,20,31]	18
478	A>C	Iran[2], Russia[4]	R[2,4]	3
479	C>A	Mexico[26], Turkey[27]	R[26,27]	2
480	TGAC insertion	Korea[6]	R	1
481	G>C	Russia[4]	R	2
484	G>A	China[63]	R	1
484 & 494	9-bp deletion & 2-bp deletion	Japan[16]	R	1
485	G>A	Brazil[17], Hong Kong[28], China[31], USA[39]	R[17,28,31,39]	5
	GT deletion	Korea[20]	R	1
486	T insertion	Poland[66]	R	1
	10-bp deletion	Japan[46]	R	1
487	10-bp deletion	Mexico[26]	R	1
488	T>C	India[50]	R	1
489	T insertion	Korea[1]	R	1
490	T>C	Brazil[11,49]	R[11,49]	2
	T deletion	Peru[32]	R	5
495	C insertion	Brazil[11]	R	1
497	1-bp insertion	Japan[16]	R	1
499	A>C	China[47]	R	1
501	CG insertion	China[15]	R	1
502	A>C	Korea[20], China[31], Netherlands[41], Spain[59]	R[20,31,41,59]	6
503	C>A	USA[12]	R	1
511	G>A	Portugal[9], Bangladesh[10], USA[33]	R[9,10,33]	3
	G>C	India[58]	R	1
512	C>A	Korea[1], Brazil[17], China[48]	R[1,17,48]	5
	C>T	China[63], Canada[64]	*R/S[63], R[64]	3
	C deletion	Rwanda[10]	R	1
513	GC deletion	Korea[7]	R	1
514 &	C>G & T>C	Belgium[10]	R	2

515				
515	T>C	Korea[6], Canada[22], Brazil[23], USA[33,39], Sweden[38,61], China[48], Cuba[56], South Africa[60]	R[6,22,23,33,338,39,48,56,60,61]	15
	T>G	Belgium[43], Poland[66]	R[43,66]	5
	T deletion	South Africa[3]	R	1
516	G insertion	Netherlands[41]	R	1
520	G>T	Korea[20]	R	2
521	A>G	China[31]	R	1
	GG insertion	Thailand[8]	R	1
	T insertion	Peru[32]	R	1
523	A>G	Japan[19], Brazil[23], Belgium[43], Poland[66]	R[19,23,43,66]	7
524	T>G	Korea[20]	R	1
	T>C	Sweden[38,61]	R[38,61]	2
525	G>A	China[31], Belgium[43], Brazil[54]	R[31,54], *R/S[43]	4
529	A>C	Taiwan[18]	R	1
530	C deletion	Belgium[43]	R	2
531	6-bp deletion	Poland[66]	R	3
532	C insertion	Korea[6]	R	1
535	A>C	Iran[2], South Africa[24]	R[2,24]	3
537	G>T	Latvia[25]	R	1
538	G>T	Korea[6], China[47], India[50], Canada[64]	R[6,47,50,64]	5
542	A deletion	China[34]	R	1
543	G>T	South Africa[24]	R	1
545	T>C	France[37]	R	1
551	G>A	Taiwan[18]	R	1
554	G>C	South Africa[24]	R	8
559	T>G	India[50]	R	1
564	G>C	Brazil[54]	R	1
572	G>A	Iran[2]	R	1
573	A>C	Canada[64]	R	1
582	T deletion	South Africa[3]	R	1

Supplementary references:

1. Kim HJ, Kwak HK, Lee J, Yun YJ, Lee JS, Lee MS, et al. Patterns of *pncA* mutations in drug-resistant *Mycobacterium tuberculosis* isolated from patients in South Korea. International Journal of Tuberculosis and Lung Disease. 2012;16(1):98-103.
2. Doustdar F, Khosravi AD, Farnia P. *Mycobacterium tuberculosis* Genotypic Diversity in Pyrazinamide-Resistant Isolates of Iran. Microbial Drug Resistance. 2009;15(4):251-6.
3. Mphahlele M, Syre H, Valvatne H, Stavrum R, Mannsaker T, Muthivhi T, et al. Pyrazinamide resistance among South African multidrug-resistant *Mycobacterium tuberculosis* isolates. Journal of Clinical Microbiology. 2008;46(10):3459-64.
4. Marttila HJ, Marjamaki M, Vyshnevskaya E, Vyshnevskiy BI, Otten TF, Vasilyef AV, et al. *pncA* mutations in pyrazinamide-resistant *Mycobacterium tuberculosis* isolates from northwestern Russia. Antimicrobial Agents and Chemotherapy. 1999;43(7):1764-6.
5. Choi J-H, Lee KW, Kang H-R, Hwang YI, Jang S, Kim D-G, et al. Clinical Efficacy of Direct DNA Sequencing Analysis on Sputum Specimens for Early Detection of Drug-Resistant *Mycobacterium tuberculosis* in a Clinical Setting. Chest. 2010;137(2):393-400.
6. Lee KW, Lee JM, Jung KS. Characterization of *pncA* mutations of pyrazinamide-resistant *Mycobacterium tuberculosis* in Korea. Journal of Korean Medical Science. 2001;16(5):537-43.
7. Park SK, Lee JY, Chang CL, Lee MK, Son HC, Kim CM, et al. *pncA* mutations in clinical *Mycobacterium tuberculosis* isolates from Korea. Bmc Infectious Diseases. 2001;1:art. no.-4.
8. Jonmalung J, Prammananan T, Leechawengwongs M, Chaiprasert A. Surveillance of pyrazinamide susceptibility among multidrug-resistant *Mycobacterium tuberculosis* isolates from Siriraj Hospital, Thailand. Bmc Microbiology. 2010;10.
9. Portugal I, Barreiro L, Moniz-Pereira J, Brum L. *pncA* mutations in pyrazinamide-resistant *Mycobacterium tuberculosis* isolates in Portugal. Antimicrobial Agents and Chemotherapy. 2004;48(7):2736-8.
10. Mestdagh M, Fonteyne PA, Realini L, Rossau R, Jannes G, Mijs W, et al. Relationship between pyrazinamide resistance, loss of pyrazinamidase activity, and mutations in the *pncA* locus in multidrug-

resistant clinical isolates of *Mycobacterium tuberculosis*. Antimicrobial Agents and Chemotherapy. 1999;43(9):2317-9.

11. Barco P, Cardoso RF, Hirata RDC, Leite CQF, Pandolfi JR, Sato DN, et al. *pncA* mutations in pyrazinamide-resistant *Mycobacterium tuberculosis* clinical isolates from the southeast region of Brazil. Journal of Antimicrobial Chemotherapy. 2006;58(5):930-5.
12. Cheng SJ, Thibert L, Sanchez T, Heifets L, Zhang Y. *pncA* mutations as a major mechanism of pyrazinamide resistance in *Mycobacterium tuberculosis*: Spread of a monoresistant strain in Quebec, Canada. Antimicrobial Agents and Chemotherapy. 2000;44(3):528-32.
13. Escalante P, Ramaswamy S, Sanabria H, Soini H, Pan X, Valiente-Castillo O, et al. Genotypic characterization of drug-resistant *Mycobacterium tuberculosis* isolates from Peru. Tubercle and lung disease : the official journal of the International Union against Tuberculosis and Lung Disease. 1998;79(2):111-8.
14. Ando H, Mitarai S, Kondo Y, Suetake T, Sekiguchi JI, Kato S, et al. Pyrazinamide resistance in multidrug-resistant *Mycobacterium tuberculosis* isolates in Japan. Clinical microbiology and infection : the official publication of the European Society of Clinical Microbiology and Infectious Diseases. 2010;16(8):1164-8.
15. Hou L, Osei-Hyiaman D, Zhang Z, Wang B, Yang A, Kano K. Molecular characterization of *pncA* gene mutations in *Mycobacterium tuberculosis* clinical isolates from China. Epidemiology and Infection. 2000;124(2):227-32.
16. Miyagi C, Yamane N, Yogesh B, Ano H, Takashima T. Genetic and phenotypic characterization of pyrazinamide-resistant *Mycobacterium tuberculosis* complex isolates in Japan. Diagnostic Microbiology and Infectious Disease. 2004;48(2):111-6.
17. Rodrigues V, Telles MA, Ribeiro MO, Cafrune PU, Rossetti MLR, Zaha A. Characterization of *pncA* mutations in pyrazinamide-resistant *Mycobacterium tuberculosis* in Brazil. Antimicrobial Agents and Chemotherapy. 2005;49(1):444-6.
18. Chiu Y-C, Huang S-F, Yu K-W, Lee Y-C, Feng J-Y, Su W-J. Characteristics of *pncA* mutations in multidrug-resistant tuberculosis in Taiwan. Bmc Infectious Diseases. 2011;11.
19. Sekiguchi J-i, Miyoshi-Akiyama T, Augustynowicz-Kopec E, Zwolska Z, Kirikae F, Toyota E, et al. Detection of multidrug resistance in *Mycobacterium tuberculosis*. Journal of Clinical Microbiology. 2007;45(1):179-92.
20. Jnawali HN, Hwang SC, Park YK, Kim H, Lee YS, Chung GT, et al. Characterization of mutations in multi- and extensive drug resistance among strains of *Mycobacterium tuberculosis* clinical isolates in Republic of Korea. Diagnostic Microbiology and Infectious Disease. 2013;76(2):187-96.
21. Bishop KS, Blumberg L, Trollip AP, Smith AN, Roux L, York DF, et al. Characterisation of the *pncA* gene in *Mycobacterium tuberculosis* isolates from Gauteng, South Africa. International Journal of Tuberculosis and Lung Disease. 2001;5(10):952-7.
22. Hirano K, Takahashi M, Kazumi Y, Fukasawa Y, Abe C. Mutation in *pncA* is a major mechanism of pyrazinamide resistance in *Mycobacterium tuberculosis*. Tubercle and Lung Disease. 1998;78(2):117-22.
23. Bhuju S, Fonseca LdS, Marsico AG, de Oliveira Vieira GB, Sobral LF, Stehr M, et al. *Mycobacterium tuberculosis* isolates from Rio de Janeiro reveal unusually low correlation between pyrazinamide resistance and mutations in the *pncA* gene. Infection Genetics and Evolution. 2013;19:1-6.
24. Louw GE, Warren RM, Donald PR, Murray MB, Bosman M, Van Heiden PD, et al. Frequency and implications of pyrazinamide resistance in managing previously treated tuberculosis patients. International Journal of Tuberculosis and Lung Disease. 2006;10(7):802-7.
25. Tracevska T, Jansone I, Baumanis V, Nodieva A, Marga O, Skenders G. Spectrum of *pncA* mutations in multidrug-resistant *Mycobacterium tuberculosis* isolates obtained in Latvia. Antimicrobial Agents and Chemotherapy. 2004;48(8):3209-10.
26. Cuevas-Cordoba B, Oyuki Xochihua-Gonzalez S, Cuellar A, Fuentes-Dominguez J, Zenteno-Cuevas R. Characterization of *pncA* gene mutations in pyrazinamide-resistant *Mycobacterium tuberculosis* isolates from Mexico. Infection Genetics and Evolution. 2013;19:330-4.
27. Yuksel P, Tansel O. Characterization of *pncA* mutations of pyrazinamide-resistant *Mycobacterium tuberculosis* in Turkey. New Microbiologica. 2009;32(2):153-8.
28. Chan RCY, Hui M, Chan EWC, Au TK, Chin ML, Yip CK, et al. Genetic and phenotypic characterization of drug-resistant *Mycobacterium tuberculosis* isolates in Hong Kong. Journal of Antimicrobial Chemotherapy. 2007;59(5):866-73.
29. Huang TS, Lee SSJ, Tu HZ, Huang WK, Chen YS, Huang CK, et al. Correlation between pyrazinamide activity and *pncA* mutations in *Mycobacterium tuberculosis* isolates in Taiwan. Antimicrobial Agents and Chemotherapy. 2003;47(11):3672-3.

30. Davies AP, Billington OJ, McHugh TD, Mitchison DA, Gillespie SH. Comparison of phenotypic and genotypic methods for pyrazinamide susceptibility testing with *Mycobacterium tuberculosis*. Journal of Clinical Microbiology. 2000;38(10):3686-8.
31. Tan Y, Hu Z, Zhang T, Cai X, Kuang H, Liu Y, et al. Role of *pncA* and *rpsA* Gene Sequencing in Detection of Pyrazinamide Resistance in *Mycobacterium tuberculosis* Isolates from Southern China. Journal of Clinical Microbiology. 2014;52(1):291-7.
32. Sheen P, Mendez M, Gilman RH, Pena L, Caviedes L, Zimic MJ, et al. Sputum PCR-Single-Strand Conformational Polymorphism Test for Same-Day Detection of Pyrazinamide Resistance in Tuberculosis Patients. Journal of Clinical Microbiology. 2009;47(9):2937-43.
33. Morlock GP, Crawford JT, Butler WR, Brim SE, Sikes D, Mazurek GH, et al. Phenotypic characterization of *pncA* mutants of *Mycobacterium tuberculosis*. Antimicrobial Agents and Chemotherapy. 2000;44(9):2291-5.
34. Huang Z-k, Luo Q, Jiang B-x, Li W-t, Xu X-m, Xiong G-l, et al. Performance of the microscopic observation drug susceptibility assay in pyrazinamide susceptibility testing for *Mycobacterium tuberculosis*. Chinese Medical Journal. 2013;126(22):4334-9.
35. Perdigao J, Macedo R, Joao I, Fernandes E, Brum L, Portugal I. Multidrug-resistant tuberculosis in Lisbon, Portugal: A molecular epidemiological perspective. Microbial Drug Resistance. 2008;14(2):133-43.
36. Endoh T, Yagihashi A, Uehara N, Kobayashi D, Tsuji N, Nakamura M, et al. Pyrazinamide resistance associated with *pncA* gene mutation in *Mycobacterium tuberculosis* in Japan. Epidemiology and Infection. 2002;128(2):337-42.
37. Lemaitre N, Sougakoff W, Truffot-Pernot C, Jarlier V. Characterization of new mutations in pyrazinamide-resistant strains of *Mycobacterium tuberculosis* and identification of conserved regions important for the catalytic activity of the pyrazinamidase *PncA*. Antimicrobial Agents and Chemotherapy. 1999;43(7):1761-3.
38. Jureen P, Werngren J, Toro J-C, Hoffner S. Pyrazinamide resistance and *pncA* gene mutations in *Mycobacterium tuberculosis*. Antimicrobial Agents and Chemotherapy. 2008;52(5):1852-4.
39. Denkin S, Volokhov D, Chizhikov V, Zhang Y. Microarray-based *pncA* genotyping of pyrazinamide-resistant strains of *Mycobacterium tuberculosis*. Journal of Medical Microbiology. 2005;54(12):1127-31.
40. Piersimoni C, Mustazzolu A, Giannoni F, Bornigia S, Gherardi G, Fattorini L. Prevention of False Resistance Results Obtained in Testing the Susceptibility of *Mycobacterium tuberculosis* to Pyrazinamide with the Bactec MGIT 960 System Using a Reduced Inoculum. Journal of Clinical Microbiology. 2013;51(1):291-4.
41. Simons SO, van Ingen J, van der Laan T, Mulder A, Dekhuijzen PNR, Boeree MJ, et al. Validation of *pncA* Gene Sequencing in Combination with the Mycobacterial Growth Indicator Tube Method To Test Susceptibility of *Mycobacterium tuberculosis* to Pyrazinamide. Journal of Clinical Microbiology. 2012;50(2):428-34.
42. Zhdanova S, Heysell SK, Ogarkov O, Boyarinova G, Alexeeva G, Pholwat S, et al. Primary Multidrug-Resistant *Mycobacterium tuberculosis* in 2 Regions, Eastern Siberia, Russian Federation. Emerging Infectious Diseases. 2013;19(10):1649-52.
43. Stoffels K, Mathys V, Fauville-Dufaux M, Wintjens R, Bifani P. Systematic Analysis of Pyrazinamide-Resistant Spontaneous Mutants and Clinical Isolates of *Mycobacterium tuberculosis*. Antimicrobial Agents and Chemotherapy. 2012;56(10):5186-93.
44. Brown TJ, Tansel O, French GL. Simultaneous identification and typing of multi-drug-resistant *Mycobacterium tuberculosis* isolates by analysis of *pncA* and *rpoB*. Journal of Medical Microbiology. 2000;49(7):651-6.
45. Pandey S, Newton S, Upton A, Roberts S, Drinkovic D. Characterisation of *pncA* mutations in clinical *Mycobacterium tuberculosis* isolates in New Zealand. Pathology. 2009;41(6):582-4.
46. Suzuki Y, Suzuki A, Tamaru A, Katsukawa C, Oda H. Rapid detection of pyrazinamide-resistant *Mycobacterium tuberculosis* by a PCR-based *in vitro* system. Journal of Clinical Microbiology. 2002;40(2):501-7.
47. Zhang H, Bi LJ, Li CY, Sun ZG, Deng JY, Zhang XE. Mutations Found in the *pncA* Gene of *Mycobacterium tuberculosis* in Clinical Pyrazinamide-resistant Isolates from a Local Region of China. Journal of International Medical Research. 2009;37(5):1430-5.
48. Zhou M, Geng X, Chen J, Wang X, Wang D, Deng J, et al. Rapid Colorimetric Testing for Pyrazinamide Susceptibility of *M. tuberculosis* by a PCR-Based *In-Vitro* Synthesized Pyrazinamidase Method. Plos One. 2011;6(11).

49. Ghiraldi LD, Campanerut PAZ, Spositto FLE, Sato DN, Leite CQF, Hirata M, et al. Evaluation of the microscopic observation drug susceptibility assay for detection of *Mycobacterium tuberculosis* resistance to pyrazinamide. Clinical Microbiology and Infection. 2011;17(12):1792-7.
50. Shenai S, Rodrigues C, Sadani M, Sukhadia N, Mehta A. Comparison of phenotypic and genotypic methods for pyrazinamide susceptibility testing. The Indian journal of tuberculosis. 2009;56(2):82-90.
51. Li H, Chen J, Zhou M, Geng X, Yu J, Wang W, et al. Rapid Detection of *Mycobacterium tuberculosis* and Pyrazinamide Susceptibility Related to *pncA* Mutations in Sputum Specimens through an Integrated Gene-to-Protein Function Approach. Journal of Clinical Microbiology. 2014;52(1):260-7.
52. Pholwat S, Stroup S, Gratz J, Trangan V, Foongladda S, Kumburu H, et al. Pyrazinamide susceptibility testing of *Mycobacterium tuberculosis* by high resolution melt analysis. Tuberculosis. 2014;94(1):20-5.
53. Aono A, Hirano K, Hamasaki S, Abe C. Evaluation of BACTEC MGIT 960 PZA medium for susceptibility testing of *Mycobacterium tuberculosis* to pyrazinamide (PZA): compared with the results of pyrazinamidase assay and Kyokuto PZA test. Diagnostic Microbiology and Infectious Disease. 2002;44(4):347-52.
54. Clemente WT, Soares Lima SS, Palaci M, Silva MSN, Sumnienski Rodrigues VF, Dalla Costa ER, et al. Phenotypic and genotypic characterization of drug-resistant *Mycobacterium tuberculosis* strains. Diagnostic Microbiology and Infectious Disease. 2008;62(2):199-204.
55. Hannan MM, Desmond EP, Morlock GP, Mazurek GH, Crawford JT. Pyrazinamide-monoresistant *Mycobacterium tuberculosis* in the United States. Journal of Clinical Microbiology. 2001;39(2):647-50.
56. Mirabal NC, Yzquierdo SL, Lemus D, Madruga M, Milian Y, Echemendia M, et al. Evaluation of Colorimetric Methods Using Nicotinamide for Rapid Detection of Pyrazinamide Resistance in *Mycobacterium tuberculosis*. Journal of Clinical Microbiology. 2010;48(8):2729-33.
57. Hoffner S, Angeby K, Sturegard E, Jonsson B, Johansson A, Sellin M, et al. Proficiency of drug susceptibility testing of *Mycobacterium tuberculosis* against pyrazinamide: the Swedish experience. International Journal of Tuberculosis and Lung Disease. 2013;17(11):1486-90.
58. Muthaiah M, Jagadeesan S, Ayalusamy N, Sreenivasan M, Prabhu SS, Muthuraj U, et al. Molecular Epidemiological Study of Pyrazinamide-Resistance in Clinical Isolates of *Mycobacterium tuberculosis* from South India. International Journal of Molecular Sciences. 2010;11(7):2670-80.
59. Aragon LM, Garrigo M, Moreno C, Espanol M, Coll P. Evaluation of the BacT/ALERT PZA kit in comparison with the BACTEC 460TB PZA for testing *Mycobacterium tuberculosis* susceptibility to pyrazinamide. Journal of Antimicrobial Chemotherapy. 2007;60(3):655-7.
60. Daum LT, Rodriguez JD, Worthy SA, Ismail NA, Omar SV, Dreyer AW, et al. Next-Generation Ion Torrent Sequencing of Drug Resistance Mutations in *Mycobacterium tuberculosis* Strains. Journal of Clinical Microbiology. 2012;50(12):3831-7.
61. Werngren J, Sturegard E, Jureen P, Angeby K, Hoffner S, Schon T. Reevaluation of the Critical Concentration for Drug Susceptibility Testing of *Mycobacterium tuberculosis* against Pyrazinamide Using Wild-Type MIC Distributions and *pncA* Gene Sequencing. Antimicrobial Agents and Chemotherapy. 2012;56(3):1253-7.
62. Lee ASG, Tang LLH, Lim IHK, Wong SY. Characterization of pyrazinamide and ofloxacin resistance among drug resistant *Mycobacterium tuberculosis* isolates from Singapore. International journal of infectious diseases : IJID : official publication of the International Society for Infectious Diseases. 2002;6(1):48-51.
63. Cui Z, Wang J, Lu J, Huang X, Zheng R, Hu Z. Evaluation of Methods for Testing the Susceptibility of Clinical *Mycobacterium tuberculosis* Isolates to Pyrazinamide. Journal of Clinical Microbiology. 2013;51(5):1374-80.
64. Alexander DC, Ma JH, Guthrie JL, Blair J, Chedore P, Jamieson FB. Gene Sequencing for Routine Verification of Pyrazinamide Resistance in *Mycobacterium tuberculosis*: a Role for *pncA* but Not *rpsA*. Journal of Clinical Microbiology. 2012;50(11):3726-8.
65. Watcharasamphankul W, Houpt ER, Foongladda S. Rapid detection of pyrazinamide resistant *Mycobacterium tuberculosis* by high resolution melting curve analysis. Journal of the Medical Association of Thailand = Chotmaihet thangphaet. 2013;96(9):1218-23.
66. Napiorkowska A, Ruesch-Gerdes S, Hillemann D, Richter E, Augustynowicz-Kopec E. Characterisation of pyrazinamide-resistant *Mycobacterium tuberculosis* strains isolated in Poland and Germany. International Journal of Tuberculosis and Lung Disease. 2014;18(4):454-60.
